# Amplification-driven BCL6-suppressed cytostasis is mediated by transrepression of FOXO3 and post-translational modifications of FOXO3 in urinary bladder urothelial carcinoma

**DOI:** 10.7150/thno.39018

**Published:** 2020-01-01

**Authors:** Wen-Ren Wu, Jen-Tai Lin, Cheng-Tang Pan, Ti-Chun Chan, Chen-Liang Liu, Wen-Jeng Wu, Jim Jinn-Chyuan Sheu, Bi-Wen Yeh, Steven K. Huang, Jheng-Yan Jhung, Meng-Shin Hsiao, Chien-Feng Li, Yow-Ling Shiue

**Affiliations:** 1 Institute of Biomedical Sciences, National Sun Yat-sen University, Kaohsiung, Taiwan.; 2 Surgery Department, Urology Division, Kaohsiung Veterans General Hospital, Kaohsiung, Taiwan.; 3 Department of Mechanical and Electro-Mechanical Engineering.; 4 Institute of Medical Science and Technology, National Sun Yat-sen University, Kaohsiung, Taiwan.; 5 Department of Medical Research, Chi Mei Medical Center, Tainan, Taiwan.; 6 Division of Urology, Department of Surgery, Chi Mei Medical Center, Tainan, Taiwan.; 7 Department of Urology, School of Medicine, College of Medicine, Kaohsiung Medical University, Kaohsiung, Taiwan.; 8 Department of Urology, Kaohsiung Medical University Hospital, Kaohsiung Medical University, Kaohsiung, Taiwan.; 9 Graduate Institute of Medicine, College of Medicine, Kaohsiung Medical University, Kaohsiung, Taiwan.; 10 Regenerative Medicine and Cell Therapy Research Center, Kaohsiung Medical University, Kaohsiung, Taiwan.; 11 Institute of Medical Science and Technology, National Sun Yat-sen University, Kaohsiung, Taiwan.; 12 Division of Urology, Department of Surgery, Chi Mei Medical Center, Tainan, Taiwan.; 13 Research Center, Faculty of Medicine Ramathibodi Hospital, Mahidol University, Bangkok, Thailand.; 14 Department of Medical Research, Chi Mei Medical Center, Tainan, Taiwan.; 15 Department of Pathology, Chi Mei Medical Center, Tainan, Taiwan.; 16 National Institute of Cancer Research, National Health Research Institutes, Tainan, Taiwan.; 17 Department of Biological Sciences, National Sun Yat-sen University, Kaohsiung, Taiwan.

**Keywords:** urinary bladder urothelial carcinoma, BCL6, FOXO3, cytostasis.

## Abstract

Muscle-invasive urinary bladder urothelial carcinoma (UBUC) is a lethal disease for which effective prognostic markers and potential therapy targets are still lacking. Previous array comparative genomic hybridization identified that 3q27 is frequently amplified in muscle-invasive UBUCs, one candidate proto-oncogene, *B-cell CLL/lymphoma 6* (*BCL6*), mapped to this region. We therefore aimed to explore its downstream targets and physiological roles in UBUC progression.

**Methods:** Specimens from UBUC patients, NOD/SCID mice and several UBUC-derived cell lines were used to perform quantitative RT-PCR, fluorescence *in situ* hybridization immunohistochemistry, xenograft, gene stable overexpression/knockdown and a series of *in vitro* experiments.

**Results:** Amplification of the *BCL6* gene lead to upregulation of *BCL6* mRNA and protein levels in a substantial set of advanced UBUCs. High BCL6 protein level significantly predicted poor disease-specific and metastasis-free survivals. Knockdown of the *BCL6* gene in J82 cells inhibited tumor growth and enhanced apoptosis in the NOD/SCID xenograft model. *In vitro* experiments demonstrated that BCL6 inhibited cytostasis, induced cell migration, invasion along with alteration of the expression levels of several related regulators. At molecular level, BCL6 inhibited *forkhead box O3* (*FOXO3*) transcription, subsequent translation and upregulation of phosphorylated/inactive FOXO3 through phosphoinositide 3-kinase (PI3K)/AKT serine/threonine kinase (AKT) and/or epidermal growth factor receptor (EGFR)/mitogen-activated protein kinase 1/2 (MAP2K1/2) signaling pathway(s). Two BCL6 binding sites on the proximal promoter region of the *FOXO3* gene were confirmed.

**Conclusion:** Overexpression of BCL6 served a poor prognostic factor in UBUC patients. *In vivo* and *in vitro* studies suggested that BCL6 functions as an oncogene through direct transrepression of the *FOXO3* gene, downregulation and phosphorylation of the FOXO3 protein.

## Introduction

Urinary bladder urothelial carcinoma (UBUC) is a common malignant disease in developed countries. Both environmental and genetic factors impact its development 1. Clinicopathological features including histological grade, stage, size and multiplicity are associated with its progression 2. Despite improvements in surgical techniques and multimodal therapy, 5-year survival rates for patients with muscle-invasive UBUC remain suboptimal 3. Almost 50% of patients eventually progress and develop systemic disease 4. Clinical and genetic heterogeneity observed in UBUC patients further complicates the use of general therapies 5. Chemotherapy is the pillar of treatment and grants survival advantages. However, due to severe side effect of chemotherapy, targeted therapy with novel drugs directed at specific molecular pathways provide novel and promising strategies to improve patient outcomes 6.

Human *B-cell CLL/lymphoma 6* (*BCL6*) gene, a transcription repressor, was originally identified through its involvement in chromosomal translocations and aberrant expression associated with B cell-derived non-Hodgkin lymphoma 7. The genome organization of the *BCL6* gene is characterized as the 5'-part encoding for Broad-complex, Tramtrack and Bric-a-brac (BTB)/POxvirus (POZ) and the 3'-end encoding for 6 DNA-binding zinc fingers 8. Upon homodimerization of BCL6 molecules, the BTB/POZ domain recruits additional corepressor molecules and forms a multi-molecular complex with nuclear receptor corepressor 2 (NCOR2, also known as SMRT), NCOR1 or BCL6 corepressor (BCOR) 9-11. The central portion of BCL6 protein is another repressor domain: RD2 12. Therefore, BCL6 interactome is massive and these complexes affect the functions of many proteins directly or indirectly.

Other than lymphoid tissues, high BCL6 protein levels were observed in a variety of epidermal neoplasms, suggesting that BCL6 may involve in morphological differentiation 13. Radically, BCL6 protein levels positively correlated with the histological grade in 47 UBUC patients 14. Oncogenic properties of BCL6 in breast 15, gallbladder 16 and ovarian 17 cancers were also reported. Several BCL6 inhibitors are under intensively investigated 9. We therefore aimed to study the correlations between BCL6 protein levels and clinicopathological features, its direct target and downstream molecular signaling pathway(s) by using an independent and larger cohort, xenograft, distinct UBUC-derived cell lines.

## Methods

### Patients, tumor materials, array-based comparative genomic hybridization, quantitative RT-PCR, fluorescence *in situ* hybridization and immunohistochemistry

The institutional review board of Chi-Mei Medical Center approved the retrospective retrieval of 295 primary UBUCs with available tissue blocks (IRB10207-001), which underwent surgery with curative intent between January 1996 and May 2004 18. To profile the copy number deviations on a genome-wide scale, 35 snap frozen UBUC specimens with a high percentage of tumor elements (> 70%) sampled from the BioBank of Chi-Mei Medical Center were examined by an expert pathologist (Li CF) and subjected to aCGH analysis (Welgene, Taipei, Taiwan). The clinical pathologic features of these patients are summarized in Supplementary Table S1. Among these, 14 and 21 were non-muscle-invasive bladder cancers (NMIBCs) and muscle-invasive bladder cancers (MIBCs), respectively. The mRNA from 52 UBUCs (28 NMIBCs; 24 MIBCs) were isolated from each fresh sample by laser capture microdissection to determine the relation between *BCL6* transcript level and UBUC progression*.* An independent cohort containing 40 fresh UBUC samples (13 NMIBCs and 27 MIBCs) was also collected for evaluating the correlation between *BCL6* and *FOXO3* mRNA levels. Quantitative RT-PCR was performed as our previous study 19 (see also Supplementary Methods). A SpectrumOrange-labeled, locus-specific laboratory-developed bacterial artificial chromosome (BAC) probe targeting *BCL6* (RP11-211G3), was used to assess the *BCL6* copies on formalin-fixed, paraffin-embedded (FFPE) sections. Another SpectrumGreen-labeled BAC probe spanning 20p12.3 (RP11-19D2) was used as the reference and evaluated as previously described 20. Rearrangement of the *BCL6* gene was detected by using Vysis LSI *BCL6* (ABR) Dual Color Break Apart Rearrangement Probe (Abbott Laboratories, Abbott Park, IL, USA).

Immunohistochemistry was performed on representative sections cut from FFPE tissues at 3-μm thickness as our previous study with a few modifications (Supplementary Methods). For immunostainings, one expert pathologist (CF Li) blinded to clinicopathological information and patient outcomes interpreted the immunostainings. A labeling index was recorded as 0~4% (0+), 5~24% (1+), 25~49% (2+), 50~74% (3+) and 75~100% (4+) of tumor cells that displayed strong nuclear staining. Cases with 3+ to 4+ and 0+ to 2+ immunoexpression were regarded as high and low levels, respectively.

### Xenograft

Animal experiments were approved (#10435) by Affidavit of Approval of Animal Use Protocol, National Sun Yet-sen University. Cells were implanted into 10 NOD/SCID mice by subcutaneous injection. J82 cells (1 × 10^7^) stably carrying either shLacZ (control) or shBCL6 were resuspended in 100 μL PBS, mixed with 100 μL matrigel (BD Biosciences, San Jose, CA, USA) and introduced into the flanks of 7-week-old male mice. Tumor diameters were measured with a digital caliper every other day and the tumor volume in mm^3^ was calculated as volume = π/6(width)^2^ × length. Whole sections from formalin-fixed xenografts were analyzed by immunohistochemistry using pertinent antibodies (Supplementary Methods).

### Chemicals, cell culture, expression plasmids and stable transfection

All chemicals unless otherwise stated were purchased from Sigma-Aldrich. UBUC-derived cell lines and culture conditions are described in the Supplementary Document. The expression vector carrying *BCL6* complete DNA (RC219007) with Myc-DDK-tag (pCMV6-BCL6) and its corresponding control pCMV6-Entry (PS100001) were obtained from Origene (Herford, Germany). PCR-based cloning was used to construct the pBCL6-HaloTag plasmid using 5'-TCCCCGCGGATGGCCTCGCCGGCTGA-3' and 5'-CCGCTCGAGGCAGGCTTTGGGGAGCTCCGGA-3' primers with *Sac* II and *Xho* I restriction sites (underlined). pHRIG-AKT1 (AKT serine/threonine kinase 1, #53583), pFOXO3 (*forkhead box O3*, #1787) and pFLAG-FOXO3(TM) (#8361, triple mutations: T32A/S253A/S315A, AKT-insensitive form) were obtained from Addgene (Watertown, MA, USA) and subcloned into the pHaloTag plasmid to generate pFOXO3-HaloTag and pFOXO3(TM)-HaloTag using the In-Fusion® HD Cloning Kit (#121416, Takara, Shiga, Japan). All constructs were sequenced verified. Cells (1 × 10^6^) were transfected with 2 μg of pCMV6-Entry, pHTC HaloTag® CMV-neo (pHaloTag, Promega, Madison, WI, USA) or target plasmid by mixing with 8 μL PolyJet™ reagent (SignaGen® Laboratories, Gaithersburg, MD, USA). Transfectants were selected with media containing 800 μg/mL of G418 (Amresco, Solon, OH, USA) for 7 days, and maintained in media with 400 μg/mL of G418 for subsequent experiments.

### Lentivirus production and stable knockdown of the *BCL6* and *FOXO3* genes

Small hairpin RNA interference (shRNAi) plasmids were inserted into the pLK0.1 vector downstream of the U6 promoter. The plasmids targeting *BCL6* (shBCL6#1/ TRCN0000013606: 5'-CCACAGTGACAAACCCTACAA-3' and shBCL6#2/TRCN00000235665: 5'-TGGACACTTGCCGGAAGTTTA-3') and *FOXO3* (shFOXO3#1/TRCN0000040098: CAGACCCTCAAACTGACACAA and shFOXO3#2/TRCN0000235491: CTTGCTCATATCCCATATAAT) were used to knockdown *BCL6* and *FOXO3* genes, respectively. shLacZ (TRCN0000072223: 5'-TGTTCGCATTATCCGAACCAT-3') served as a negative control.

### *In vitro* and immunoblot assays

Flow cytometric, bromodeoxyuridine, cell proliferation, soft agar, anchorage-independent growth, wound-healing, transwell migration, transwell invasion and RNA expression profiling with the HumanHT-12v4 Expression BeadChip (Illumina, San Diego, CA, USA) assays were used to determine the alterations of cell cycle distribution, cell proliferation, colony formation/anchorage-independent cell growth, cell migration, cell invasion and mRNA levels after overexpression, knockdown of the *BCL6*, *FOXO3* gene or treatments with inhibitors, respectively 18, 19 (Supplementary Methods). KEGG mapper (https://www.genome.jp/kegg/mapper.html) was applied to identify significant pathways of the RNA expression profile from the stable *BCL6*-knockdown BFTC905 cells. Matrix metallopeptidase 2 (MMP2)/MMP9 Solution Assay Kit (#E-118SA, Biomedical Research Service Center, University at Buffalo, State University of New York, USA) was used to measure MMP2/MMP9 activity. Immunoblot analyses were performed as in our previous study to detect the expression levels of tumor protein p53 (TP53), phosphor-TP53 (serine 15) [pTP53(S15)], pTP53(S20), cyclin-dependent kinase inhibitor 1A (CDKN1A), CDKN1B, CDKN1C, CDKN2B, CDKN2C, CDKN2D, cyclin D1 (CCND1), cyclin-dependent kinase 4 (CDK4), CCNE1, CDK2, RB transcriptional corepressor 1 (RB1), E2F transcription factor 1 (E2F1), transcription factor Dp-1 (TFDP1), glyceraldehyde-3-phosphate dehydrogenase (GAPDH), cadherin 1 (CDH1), vimentin (VIM), CD44 molecule, Indian blood group (CD44), FOXO3, AKT, pAKT1(S473), mitogen-activated protein kinase 1/3 (MAPK1/3, also known as ERK1/2), pMAPK1/3(T202/Y204), pFOXO3(S253), pFOXO3(S294), HRas proto-oncogene, GTPase (HRAS) and proteins phosphatase and tensin homolog (PTEN) (Supplementary Methods).

### Luciferase reporter, chromatin immunoprecipitation and quantitative assays

Two *FOXO3* proximal promoter regions (1.2 Kb: -1100 to +100; 660 bp: -560 to +100) linked to the luciferase reporter gene plasmid (pGL4.17-A and pGL4.17-B) were PCR-cloned using genomic DNA from T24 cells. Each reporter plasmid (1000 ng) and *Renilla* luciferase plasmid (100 ng, internal control) were cotransfected into T24 cells (2 × 10^5^). After transfection for 48 h, cell lysates were prepared and subjected to Dual-luciferase Reporter Assay (Promega). Luciferase activities were normalized to *Renilla* luciferase activities. The BCL6-binding sites on the *FOXO3* promoter region were PCR-amplified from total DNA (input) and immunoprecipitated DNA (anti-BCL6) for 30 cycles. Primers spanning -911 to -906 (site 1) and -540 to -535 (site 2) were used. Quantitative chromatin immunoprecipitation (ChIP) assay was performed by using immunoprecipitated DNA and TaqMan quantitative PCR (Supplementary Methods).

### Statistics

All calculations were performed using SPSS 14.0 software (IBM, Armonk, NY, USA). The survival difference of the two clusters was calculated by log-rank analysis and plotted using the Kaplan-Meier method for survivals. The correlation, comparison between various clinicopathological factors and BCL6, pFOXO3(S253) and pFOXO3(S294) expression levels were assessed by the Chi-square test. The endpoint analyzed was disease-specific and/or metastasis-free survival. For all analyses, two-sided tests of significance were used. Normal distributed data were expressed as means ± SEM. Differences between two groups were analyzed by the Student's *t*-test. Differences among ≥3 groups were analyzed be one-way analysis of variance, followed by Scheffe multiple comparison test. A *P* < 0.05 is considered to be statistical significance.

## Results

### High BCL6 protein levels predict poor outcomes in UBUC patients

**Figure 1:**
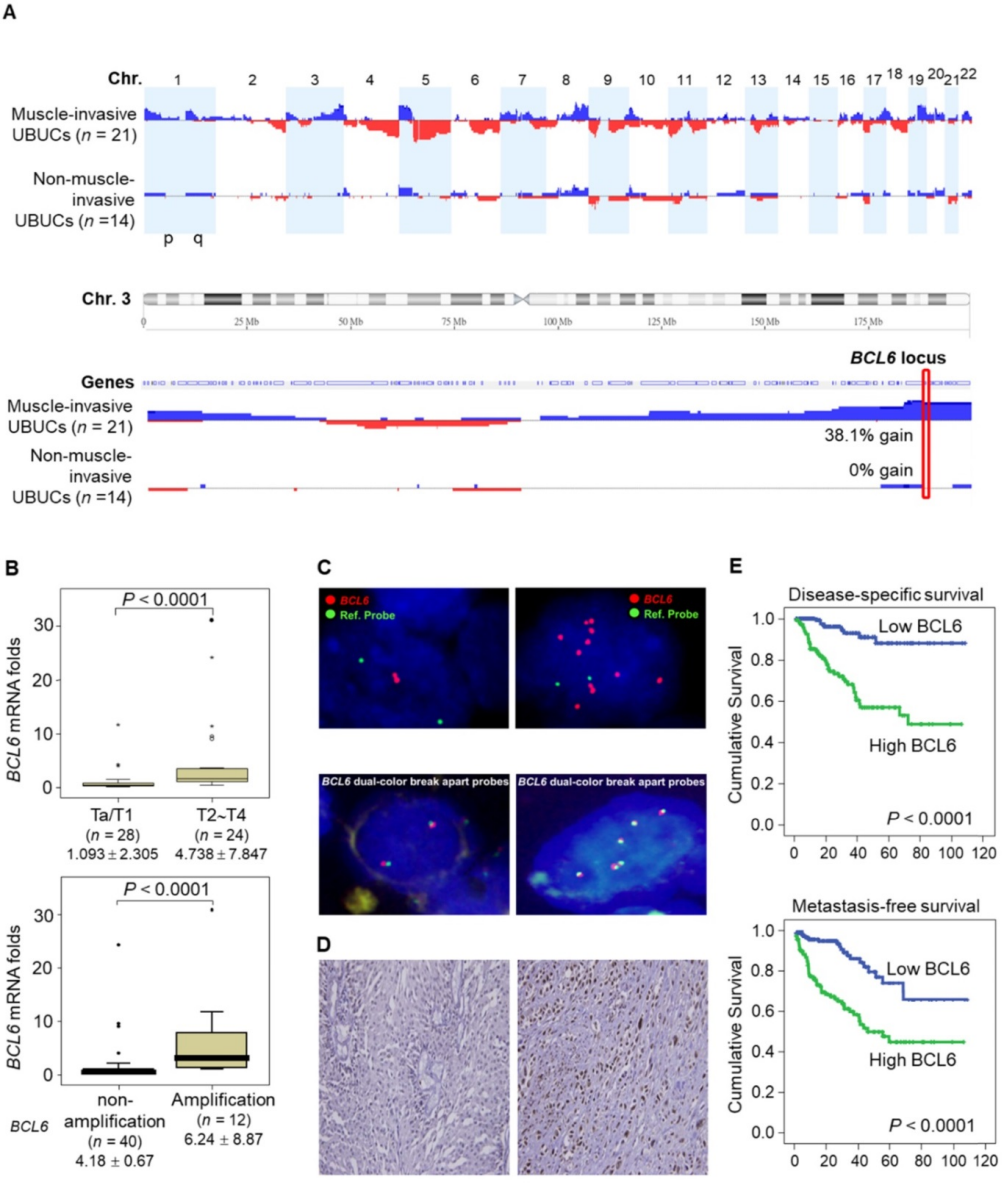
** High BCL6 protein level confers poor disease-specific and metastasis-free survivals in UBUCs. (A)** Genome-wide copy number imbalances in muscle-invasive and non-invasive UBUCs are profiled. By applying Nexus Copy Number™ software, DNA copy number gains (blue bars) and losses (red bars) in UBUCs are shown in the upward and downward directions, respectively, along the horizontal coordinate of individual chromosomes. Altered copy numbers in each autosome between muscle-invasive and non-invasive UBUCs (upper panel) are shown. Amplified region in 3q harboring the *BCL6* locus was identified exclusively in muscle-invasive UBUCs.** (B)** The *BCL6* mRNA expression folds in the pure UBUC cells from 52 fresh samples were significantly correlated with T2-T4 stage and *BCL6*-amplificated patients. **(C)** Fluorescent *in situ* hybridization and immunohistochemistry displayed representative of non-invasive UBUCs without *BCL6* amplification, rearrangement (Red: *BCL6*; Green: reference) and low BCL6 protein level **(left panel, C)**, while *BCL6* amplification without rearrangement was observed in a muscle-invasive UBUC with diffuse and strong BCL6 staining **(right panel, C)**. **(D)** High BCL6 protein level (*n* = 295) significantly predicted poor disease-specific and metastasis-free survivals.

aCGH identified that human chromosome 3q, 5p, 8q and 19q were frequently amplified (blue bars) while 2q, 4q, 5q, 6q, 9p, 9q, 11p, 11q, 13p 17p and 18q were regularly deleted (red bars) in muscle-invasive UBUC patients (upper panel, Figure 1A). Among these regions, we focused on 3q. One proto-oncogene *BCL6*, mapped to 3q27, was exclusively identified in muscle-invasive UBUCs (lower panel, Figure 1A). The *BCL6* mRNA was significantly upregulated in high pT (T2-T4) and *BCL6*-amplified compared to low pT (Ta/T1) (*P* < 0.0001) and non-amplified cases (*P* < 0.001) (Figure 1B). The *BCL6* locus was amplified but not rearranged in muscle-invasive (right panel; Figure 1C) compared to non-invasive UBUCs (left panel; Figure 1C). Strong BCL6 immunointensity in one representative invasive (right panel) compared to a non-invasive tissue (left) are shown (Figure 1D). In 295 UBUC patients, high BCL6 protein levels were significantly correlated with high pT, nodal metastasis, histological grade, vascular invasion, perineural invasion and mitotic rate (Supplementary Table S2). High BCL6 protein level significantly predicted poor survivals (Figure 1E). In univariate analysis, high pT, nodal metastasis, histological grade, vascular invasion, perineural invasion, mitotic rate and BCL6 protein levels were significantly correlated with inferior disease-specific and metastasis-free survivals. In multivariate analysis, high pT, mitotic rate and BCL6 protein level play significant and independent prognostic factors for disease-specific and metastasis-free survivals (Table 1). Therefore, *BCL6* gene amplification causes *BCL6* mRNA and protein upregulation and confer poor prognosis in UBUC patients.

### Knockdown of the *BCL6* gene suppresses tumor growth and enhances apoptosis *in vivo*

**Figure 2:**
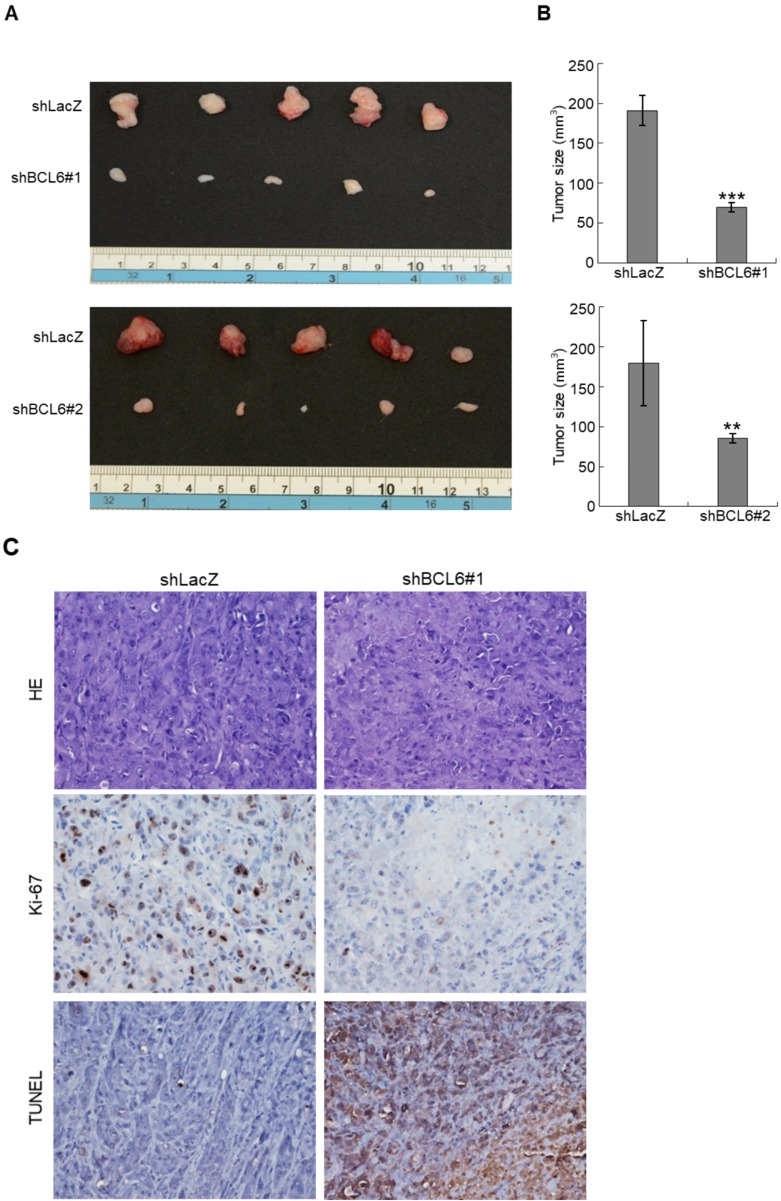
Stable knockdown of the *BCL6* gene in J82 cells inhibits tumor growth and apoptosis in the NOD/SCID xenograft model. Two distinct shRNAi clones (shBCL6#1 and shBCL6#2) was used to stable knockdown of the *BCL6* gene in J82 cells. **(A, B)** The average tumor volume of control xenografts (shLacZ) was larger than the *BCL6-*knockdown counterpart by the end of animal experiments (Day 35). **(C)** After the mice were sacrificed, shLacZ xenografts displayed a carcinoma with high cellularity, while the *BCL6-*knockdown group showed large areas of necrosis and a much lower percentage of viable components. Immunohistochemical and terminal deoxynucleotidyl transferase dUTP nick end labeling (TUNEL) assays showed that the Ki-67 protein was downregulated and apoptotic cells were increased after knockdown of the *BCL6* gene in one representative xenograft tissue. HE: hematoxylin and eosin staining. Statistical significance: **P < 0.01 and ***P < 0.001.

The *BCL6* mRNA and BCL6 protein were highly expressed in RT4, BFTC905, BFTC909, J82 and/or T24 compared to normal HUC cell line (Supplementary Figure S1A, S1B). Stable overexpression of the *BCL6* gene in J82 and/or T24; knockdown in J82, BFTC905, BFTC909 cells significantly upregulated and downregulated *BCL6* mRNA and protein levels, respectively (Supplementary Figure S1C, S1D). In NOD/SCID mice, xenografts with stable *BCL6*-knockdown J82 cells with 2 distinct shRNAi clones showed significantly smaller tumors compared to the control shLacz (Figure 2A, 2B). Stable knockdown of the *BCL6* gene markedly downregulated Ki-67 abundance while increased DNA fragmentation as indicated by the TUNEL assay compared to the control (Figure 2C), suggesting that BCL6 promotes cell proliferation and inhibits apoptosis in xenograft tissues.

### Stable *BCL6* overexpression induces while knockdown suppresses cell proliferation, cell cycle progression, colony formation/anchorage-independent cell growth, cell migration and invasion in UBUC-derived cells

**Figure 3:**
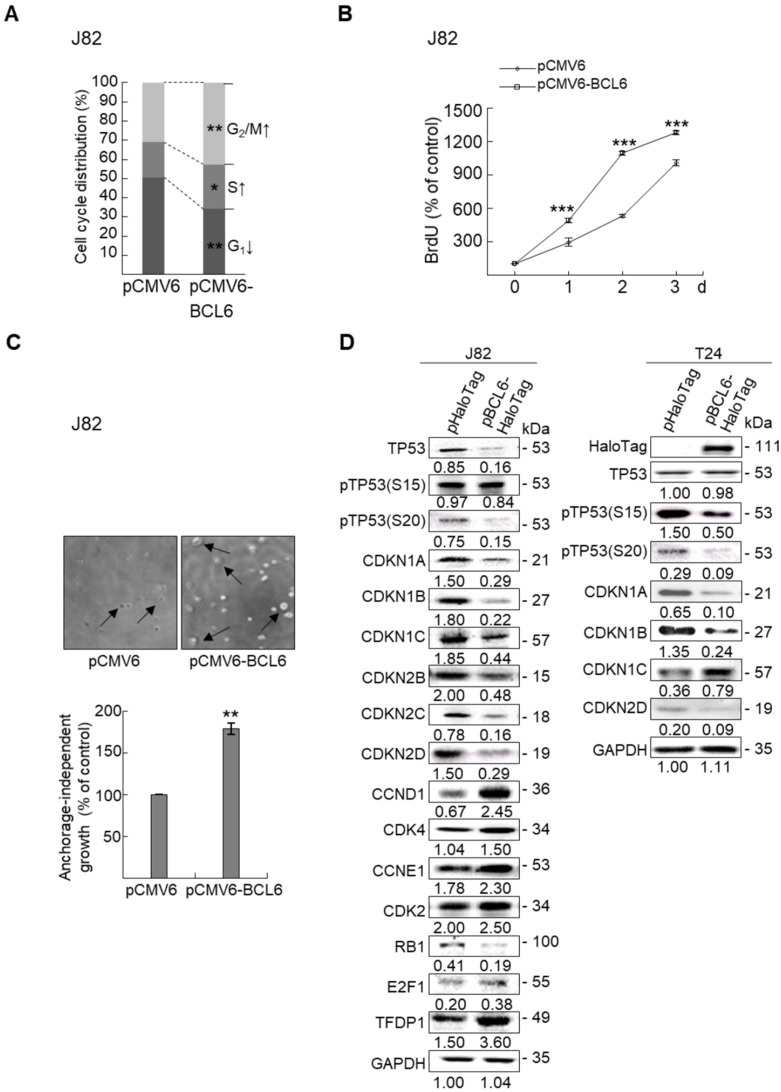

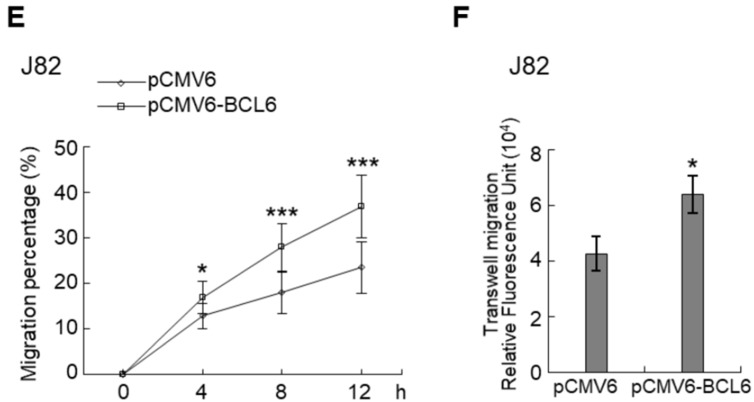

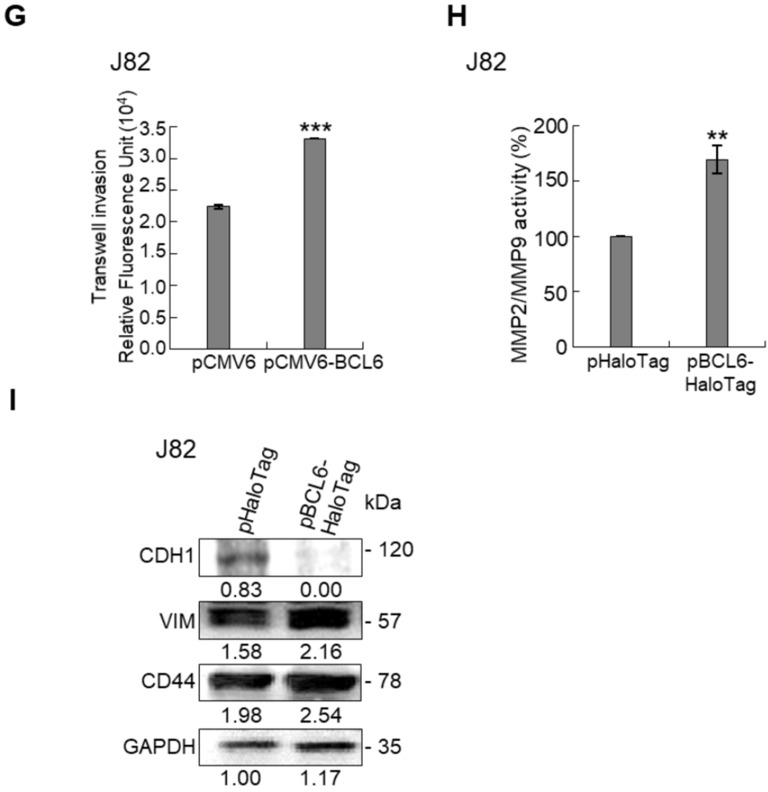

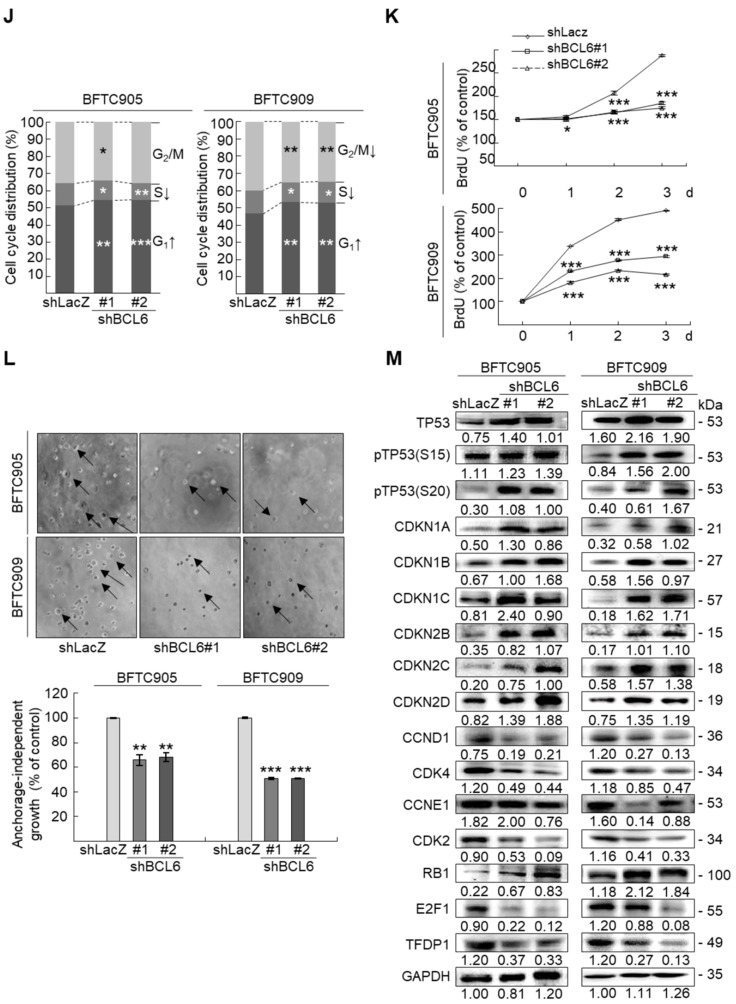

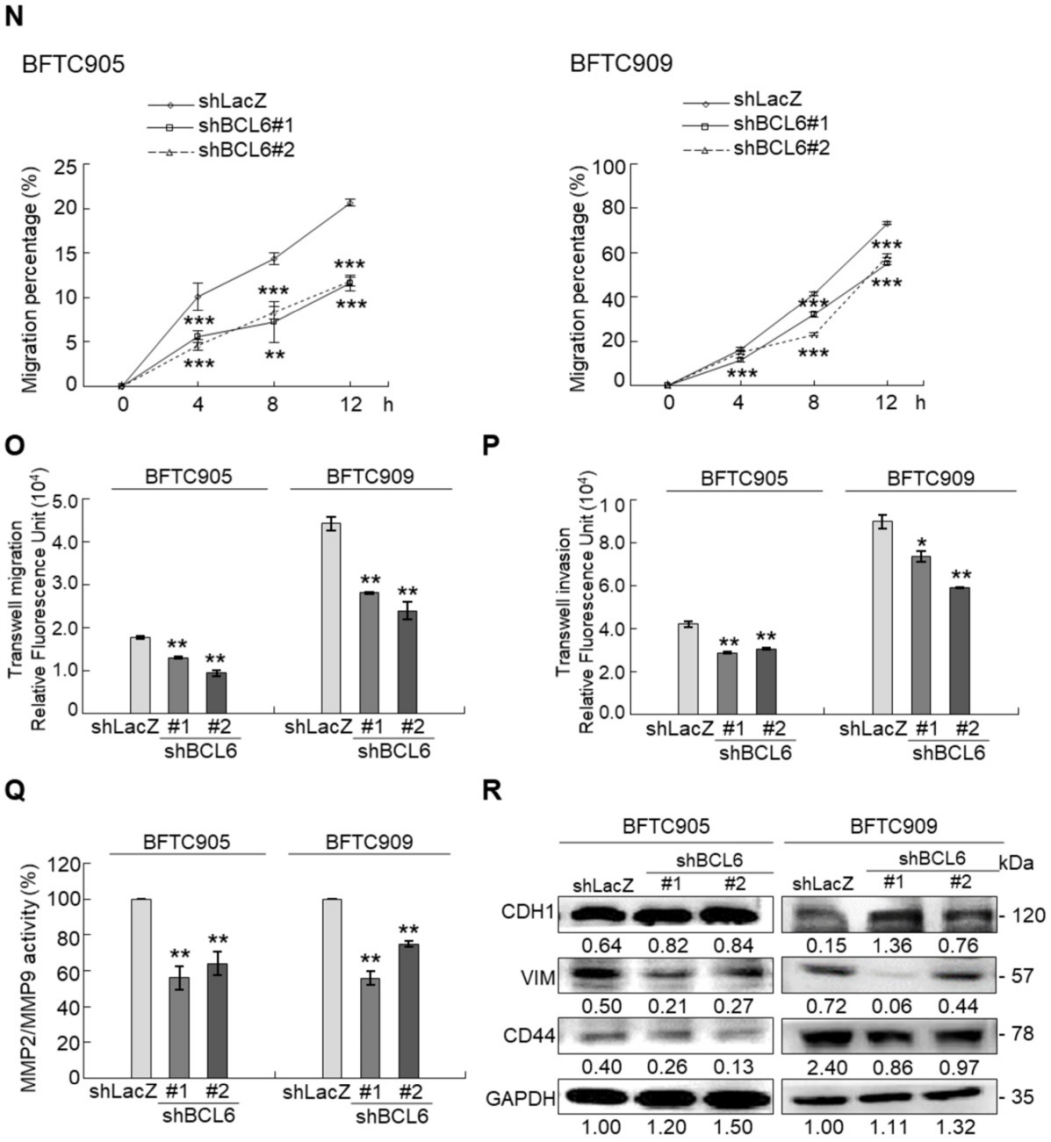
** Overexpression of the *BCL6* gene induces while knockdown suppresses cell cycle progression, anchorage-independent cell growth,** cell migration and invasion in distinct UBUC-derived cells. Flow cytometric, bromodeoxyuridine (BrdU), soft agar/3-(4,5-dimethylthiazol-2-yl)-2,5-diphenyltetrazolium bromide (MTT), transwell migration, transwell invasion, immunoblot and matrix metalloproteinase 2 (MMP2)/MMP9 activity assays identified that stable transfection of the pCMV6-BCL6 plasmid into J82 cells **(A, B)** induced **(A)** cell cycle progression from G_1_ to S and G_2_/M phases, **(B)** cell proliferation, **(C)** colony formation (arrow) and anchorage-independent cell growth, **(D)** upregulated CCND1, CDK4, CCNE1, CDK2, E2F1 and TFDP1 protein levels compared to the control (pHaloTag transfectant), however, notably downregulated TP53, pTP53(S15), pTP53(S20), CDKN1A, CDKN1B, CDKN1C, CDKN2B, CDKN2C, CDKN2D and RB1 protein levels; **(E)** cell migration from 0 to 12 h after seeding which was identified by wound-healing assay (Supplementary Methods & Fig. S3), **(F)** transwell migration,** (G)** transwell invasion, **(H)** MMP2/MMP9 activity and **(I)** downregulated CDH1 and upregulated VIM and CD44 protein levels. In stable *BCL6*-overexpressed T24 cells, pTP53(S15), pTP53(S20), CDKN1A, CDKN1B and CDKN2D protein levels were also downregulated **(D)**. On the other hand, Sable knockdown of the *BCL6* gene with two distinct shRNAi clones (shBCL6#1, shBCL6#2) in BFTC905 and BFTC909 cells exhibited the opposite phenotypes and protein expression patterns compared to *BCL6*-overexpressed J82 and/or T24 cells **(J-R)**. All experiments were performed in triplicate and results are expressed as means ± SEM. Except for wound-healing, other experiments were conducted after seeding for 48 h. For soft agar and immunoblot analyses, representative images are shown. GAPDH served as a loading control for immunoblot analysis. Statistical significance: *P < 0.05, **P < 0.01 and ***P < 0.001.

Overexpression of the *BCL6* gene in J82 and cells induced cell cycle progression to S and G_2_/M phases (*P* < 0.05, Figure 3A, see also Supplementary Figure S2), increased cell proliferation (*P* < 0.001, Figure 3B), colony formation/anchorage-independent cell growth (*P* < 0.01, Figure 3C), notably altered the expression levels of several cell cycle regulatory proteins compared to the control (Figure 3D) a similar expression pattern was also identified in *BCL6*-overexpressed T24 cells), increased cell migration by wound-healing assay (Supplementary Figure S3) from 4-12 h after seeding (Figure 3E), transwell migration and transwell invasion (Figure 3F, 3G, Supplementary Figure S4), gelatin zymography (Supplementary Figure S5) and MMP2/MMP9 activity (Figure 3H); downregulated CDH1 while upregulated VIM and CD44 protein levels (Figure 3I). On the other hand, stable knockdown of the *BCL6* gene in BFTC905 and BFTC909 cells showed opposite phenotypes and protein expression profiles to those in J82 and/or T24 cells (Figure 3J-3R, Supplementary Figure S2, S3, S4, S5). Quantitative RT-PCR next indicated that BCL6 negatively regulated *TP53* and several cyclin-dependent kinase inhibitors (CKIs) including *CDKN1A*, *CDKN1B*, *CDKN1C* and *CDKN2D* mRNA levels in J82, T24, BFTC905 and/or BFTC909 cells (Supplementary Figure S6).

### BCL6-suppressed cytostasis through transrepression of the *FOXO3* gene in UBUC-derived cells

**Figure 4:**
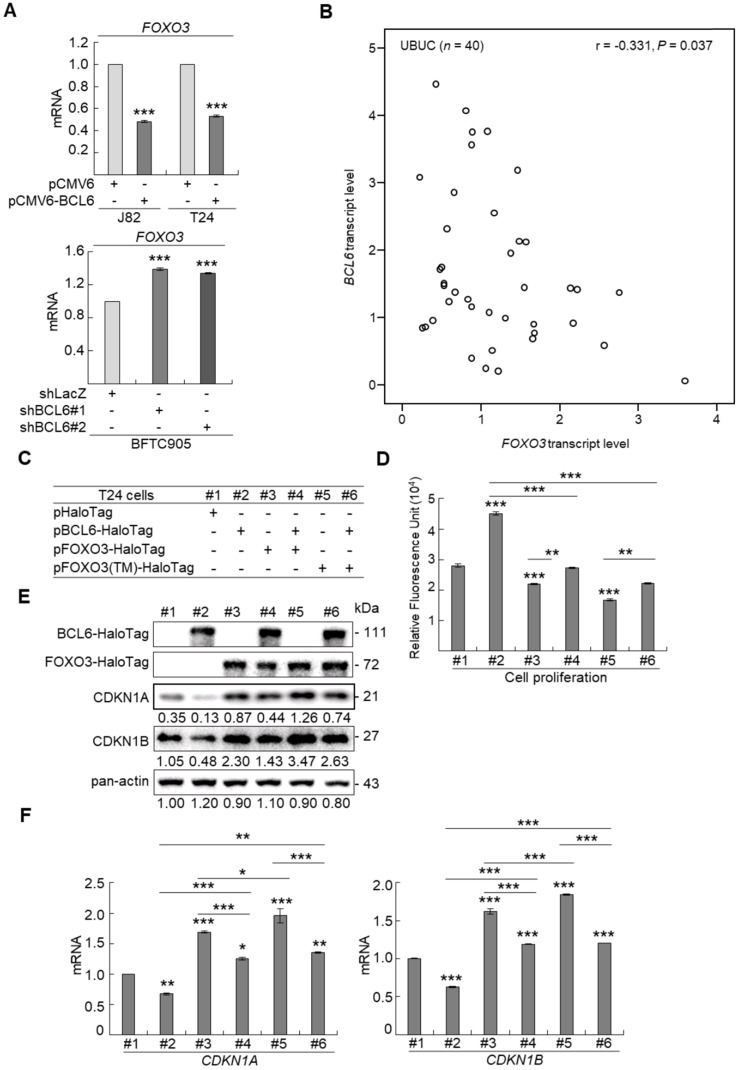

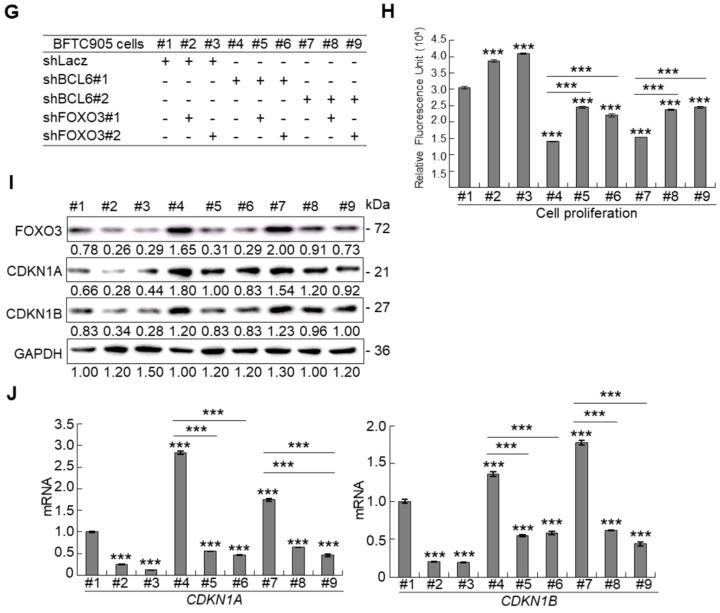

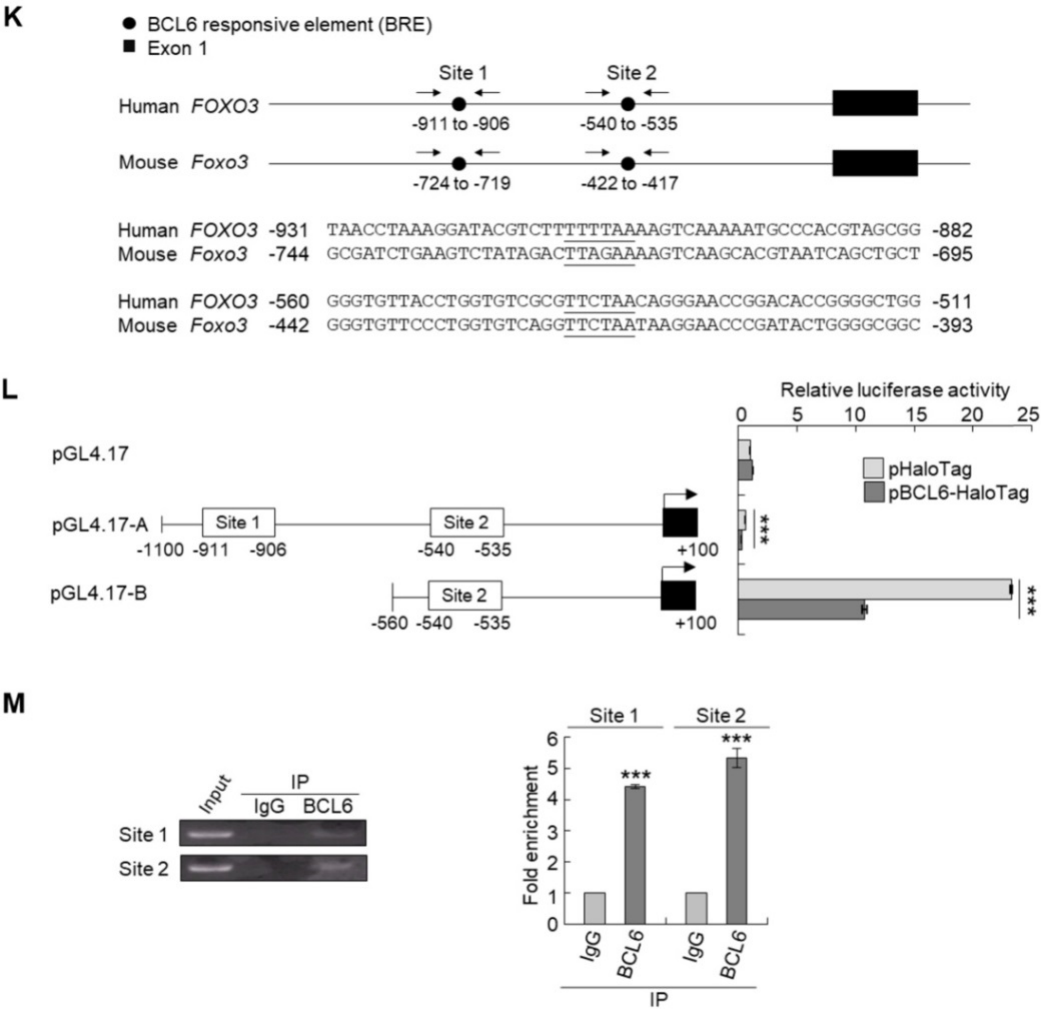
** BCL6 suppresses cytostasis through transrepression of the *FOXO3* gene.** Quantitative RT-PCR showed that transfection of the pCMV6-BCL6 plasmid into 3 × 10^5^ of J82 and T24 cells (6-cm dishes) for 48 h downregulated while stable knockdown of *BCL6* gene with 2 distinct shRNAi clones (shBCL6#1 and shBCL6#2) in BFTC905 cells (8 × 10^5^) upregulated *FOXO3* mRNA levels **(A)**, *BCL6* mRNA was negatively correlated with *FOXO3* mRNA level in 40 UBUC specimens **(B)**. **(C)** T24 cells (3 × 10^5^) were seeded overnight in 6-cm dishes and transfected with pHaloTag (control), pBCL6-HaloTag, pFOXO3-HaloTag and/or pFOXO3(TM)-HaloTag [triple mutation (T32A/S253A/S315A): AKT1-insensitive form] plasmids for 48 h. Cell proliferation, immunoblot analysis, quantitative RT-PCR showed that transfection of the pFOXO3-HaloTag (#3 vs. #1) or the pFOXO3(TM)-HaloTag (#5 vs. #1) plasmid into T24 cells for 48 h suppressed cell proliferation compared to the control (pHaloTag)** (D)**, upregulated CDKN1A and CDKN1B protein **(E)**; *CDKN1A* and *CDKN1B* mRNA **(F)** levels, respectively, compared to the control. **(G)** Co-transfection of the pBCL6-HaloTag and the pFOXO3-HaloTag (#4 vs. #3) or pFOXO3(TM)-HaloTag (#6 vs. #5) plasmids into T24 cells for 48 h restored FOXO3- and FOXO3(TM)-suppressed cell proliferation** (H)**, suppressed FOXO3- and FOXO3(TM)-induced CDKN1A, CDKN1B protein **(I)**, *CDKN1A* and *CDKN1B* mRNA** (J)** levels compared to those of the pBCL6-HaloTag transfectants.** (G)** Single and double knockdown of the *BCL6* and/or *FOXO3* gene were performed. Stable knockdown of the *FOXO3* gene with shFOXO3#1 or shFOXO3#2 in BFTC905 cells (8 × 10^5^) induced cell proliferation** (H)**, downregulated FOXO3, CDKN1A, CDKN1B protein** (I)**, *CDKN1A* and *CDKN1B* mRNA** (J)** levels compared to the control (#2 vs. #1; #3 vs. #1). Stable knockdown of the *FOXO3* gene with shFOXO3#1 or shFOXO3#2 in 2 stable *BCL6*-knockdown cell lines (#5 vs. #4, #6 vs. #4 and #8 vs. #7, #9 vs. #7) increased cell proliferation** (H)**, downregulated FOXO3, CDKN1A, CDKN1B protein **(I)**, *CDKN1A* and *CDKN1B* mRNA **(J)** levels compared to shBCL6#1 or shBCL6#2 group. **(D)** Phylogenetic footprinting technology identified two conserved BCL6 responsive elements (site 1 & site 2) in the *FOXO3* proximal promoter region across human and mouse **(K)**. Two *FOXO3* promoter regions were cloned as pGL4.17-A (-1100 to +100) and pGL4.17-B (-560 to +100) as indicated. Transcription start site (TSS) is defined as +1. Dual luciferase® reporter assay showed that transfection of the pBCL6-HaloTag plasmid for 48 h downregulated the promoter activities of pGL4.17-A (from 0.5867 ± 0.0164 to 0.2761 ± 0.0032) and pGL4.17-B activities compared to the control (pGL4.17) **(L)**. Chromatin immunoprecipitation assay, PCR and quantitative PCR identified that BCL6 bond to site 1 and site 2. IgG was used as negative control **(M)**. All experiments were performed in triplicate and results are expressed as means ± SEM. For immunoblot and PCR analyses, representative images are shown. GAPDH served as a loading control in immunoblot analysis. Statistical significance: *P < 0.05, **P < 0.01 and ***P < 0.001.

Profiling of mRNA with the BeadChip identified 16 transcripts including *FOXO3*, *CDKN1A* and *CDKN1B* (data not shown) that involved in the FOXO signaling pathway were upregulated in *BCL6*-knockdown BFTC905 cells. Quantitative RT-PCR further confirmed that BCL6 negatively regulated *FOXO3* mRNA level using different cell lines through *BCL6*-overexpressed and -knockdown experiments (Figure 4A). The correlation coefficient between *BCL6* and *FOXO3* mRNA level was -0.331 (*P* = 0.037) (Figure 4B), highly similar to those (GSE13507) deposited in the Gene Expression Omnibus database (Supplementary Fig. S7). To evaluate whether FOXO3 plays a crucial role in BCL6-suppressed cytostasis, overexpression of the *BCL6*, wild type *FOXO3* or *FOXO3(TM)* (triple mutations: T32A/S253A/S315A, AKT1-insensitive form) 21 gene (Figure 4C) along with single or double knockdown of the *BCL6* and *FOXO3* gene were performed *in vitro*. The effect of *FOXO3(TM)* gene overexpression would demonstrate whether AKT1 played a role in *BCL6*-induced cell proliferation. Overexpression of the *FOXO3* or *FOXO3(TM)* gene in T24 cells suppressed cell proliferation (*P* < 0.001, *P* < 0.001; Figure 4D), upregulated CDKN1A, CDKN1B protein (Figure 4E) and their corresponding mRNA (*P* < 0.001 for all, Figure 4F) levels. Simultaneously overexpression of the *FOXO3* and *BCL6* genes or *FOXO3(TM)* and *BCL6* genes restored FOXO3- and FOXO3(TM)-suppressed cell proliferation (*P* < 0.01, *P* < 0.01, Figure 4D), downregulated CDKN1A, CDKN1B protein (Figure 4E) and their corresponding mRNA (*P* < 0.001 for all, Figure 4F) levels. However, the effects of *FOXO3(TM)* in cytostasis, upregulation of CDKN1A, CDKN1B protein and their corresponding mRNA levels were similar to those of the *FOXO3* gene in BCL6-overexpessed T24 cells (*P* > 0.05), suggesting that both total FOXO3 and AKT1-insensitive FOXO3 play equally critical role in BCL6-suppressed cytostasis.

Knockdown of the *FOXO3* gene (Figure 4G) showed that shFOXO3#1 (*P* < 0.001) and shFOXO3#2 (*P* < 0.001) increased cell proliferation compared to the control group (shLacz). Double knockdown of the *BCL6* and *FOXO3* genes demonstrated that shFOXO3#1 (*P* < 0.001; *P* < 0.001) and shFOXO3#2 (*P* < 0.001; *P* < 0.001) restored shBCL6#1- and shBCL6#2-suppressed cell proliferation (Figure 4H), downregulated CDKN1A, CDKN1B protein (Figure 4I) and their corresponding mRNA (Figure 4J) levels (*P* < 0.001 for all) compared to shBCL6#1 or shBCL6#2 group. Therefore, BCL6 negatively regulated *FOXO3*, *CDKN1A* and *CDKN1B* at the mRNA and protein levels, leading to cell proliferation in UBUC-derived cells.

Phylogenetic footprinting technology along with searching on Eukaryotic Promoter Database 22 identified two conserved BCL6 responsive element across human and mouse genomic sequences in the proximal promoter region of the *FOXO3*/*Foxo3* gene (Figure 4K). Dual-Luciferase® reporter assay next indicated that transfection of the pBCL6-HaloTag into T24 cells for 48 h in reduced promoter activities in pGL4.17-A (Site 1: -1100 to +100) and pGL4.17-B (Site 2: -560 to +100) transfectants (*P* < 0.001) (Figure 4L). ChIP and quantitative ChIP assays validated that BCL6 direct interacts with Site 1 and Site 2 of the *FOXO3* promoter region (Figure 4M). Accordingly, BCL6 is a transsuppressor of the *FOXO3* gene.

### BCL6 regulates FOXO3-CKIs through MAPK1/3 and/or PI3K-AKT signaling pathway in UBUC-derived cells

**Figure 5:**
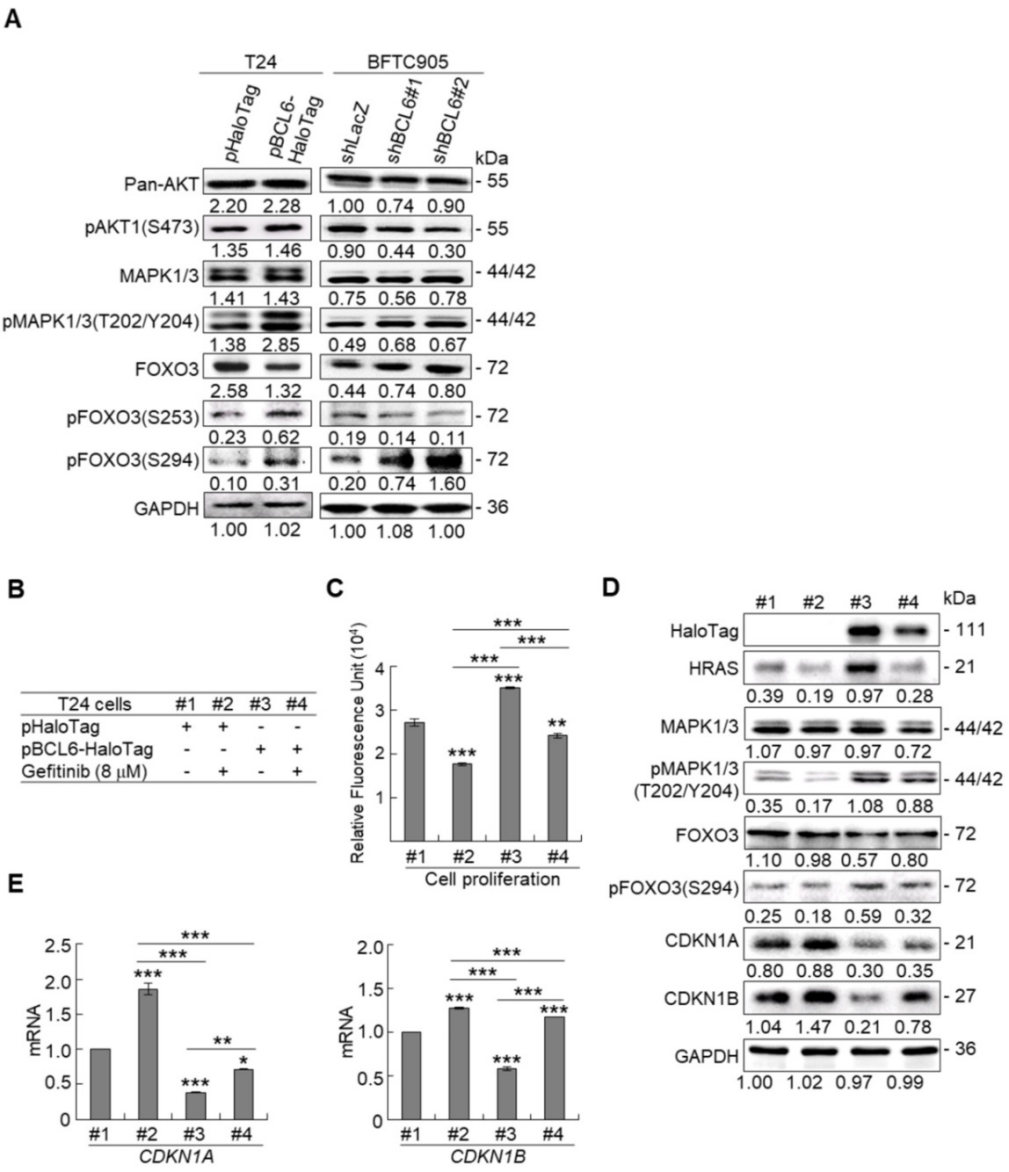

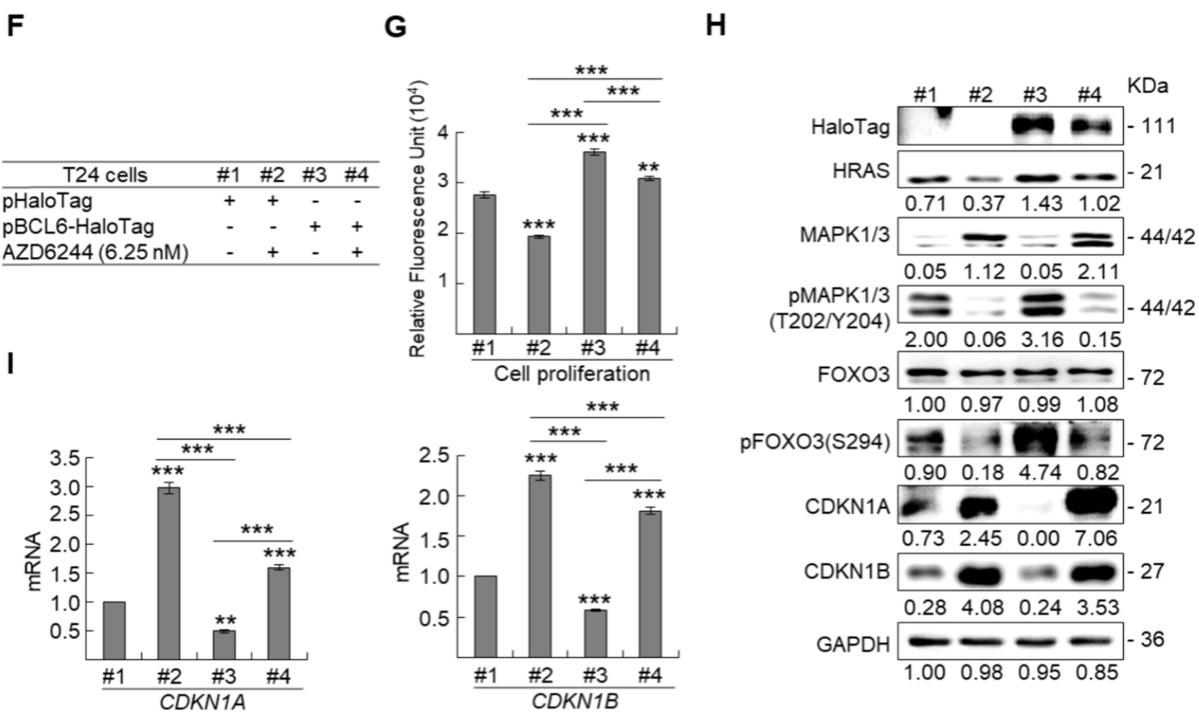

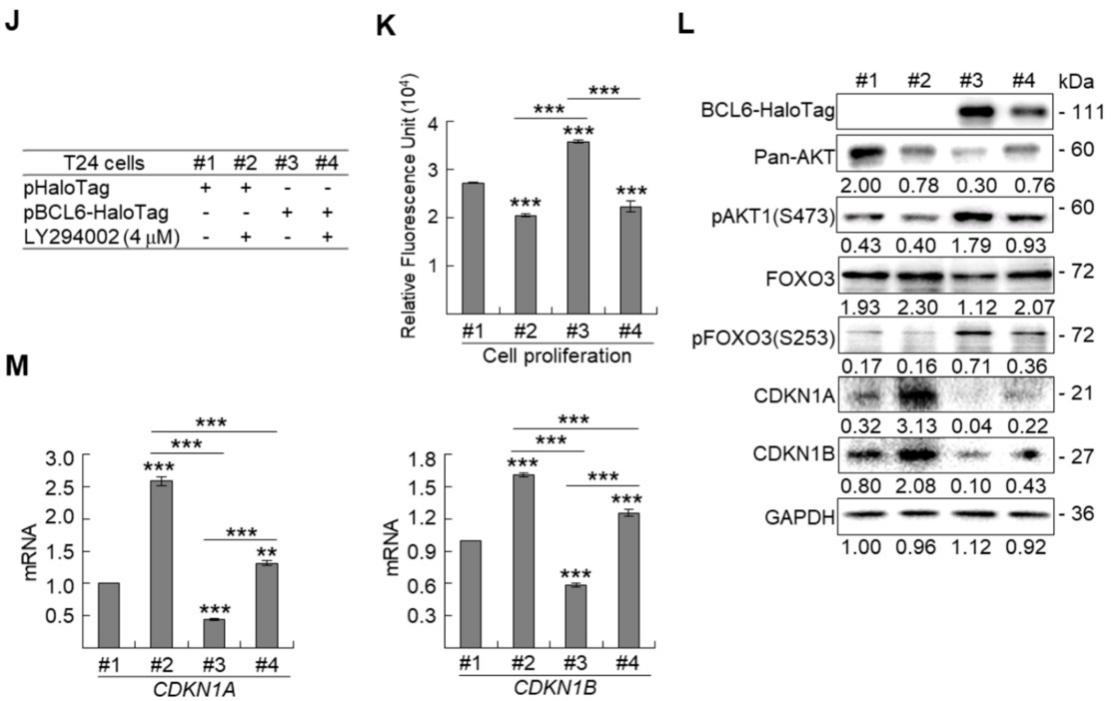

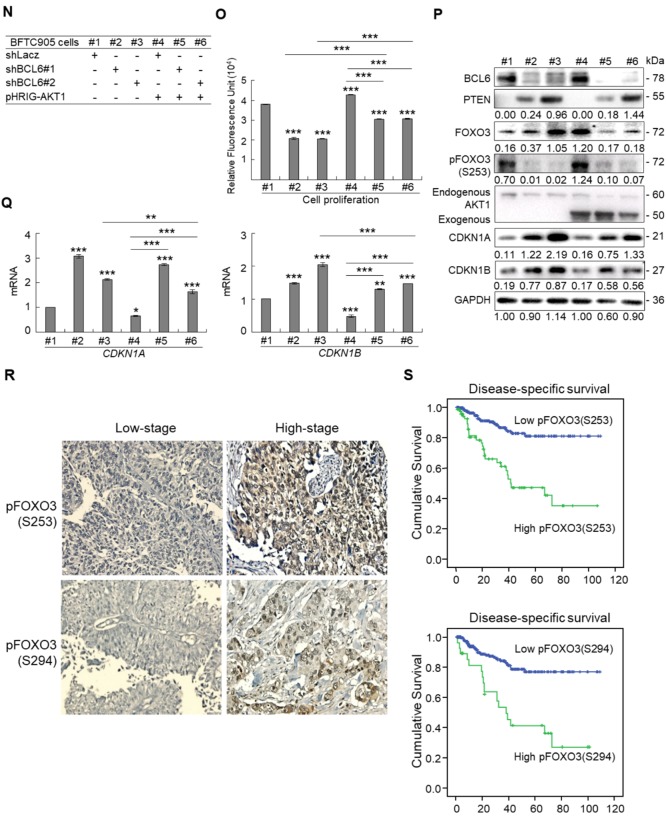
** BCL6 regulates FOXO3-CKIs through EGFR/MAP2K and/or PI3K-AKT signaling pathway in UBUC-derived cells.** Immunoblot, cell proliferation, quantitative RT-PCR assays and inhibitor treatments were performed. **(A)** Transfection of the pBCL6-HaloTag plasmid into T24 (3 × 10^5^) for 48 h upregulated pAKT1(S473), pMAPK1/3(T202/Y204), pFOXO3(S253) and pFOXO3(S294), while stable knockdown of the *BCL6* gene in BFTC905 cells (8 × 10^5^) with 2 distinct shRNAi clones downregulated pAKT1(S473) but upregulated pMAPK1/3(T202/Y204) and pFOXO3(S294) protein levels. **(B-D)** T24 (3 × 10^5^) and were seeded overnight, transfected with pHaloTag (control) or pBCL6-HaloTag plasmid for 24 h and treated with different inhibitors for another 24 h. Treatment with an EGFR inhibitor, gefitinib** (B)**, suppressed endogenous (#2 vs. #1) and BCL6-induced (#4 vs. #3) cell proliferation** (C)**, downregulated endogenous and BCL6-induced HRAS, pMAPK1/3(T202/Y204), pFOXO3(S294), while upregulated endogenous and BCL6-suppressed CDKN1A and CDKN1B protein **(D)**; *CDKN1A* and *CDKN1B* mRNA **(E)** levels. **(F-I)** Treatment with a MAP2K1/2 inhibitor, AZD6244 in T24 cells showed similar effects compared to those of treatments with gefitinib. **(J)** Treatment with a PI3K inhibitor, LY294002, in T24 cells repressed endogenous (#2 vs. #1) and BCL6-induced (#4 vs. #3) cell proliferation **(K)**, downregulated endogenous and BCL6-induced pAKT1(S473) and pFOXO3(S253) while upregulated endogenous and BCL6-suppressed CDKN1A and CDKN1B protein **(L)**; *CDKN1A* and *CDKN1B* mRNA** (M)** levels. **(N)** Transfection of a constitutively active AKT1 plasmid (pHRIG-AKT1) in stable *BCL6*-knockdown BFTC905 cells (8 × 10^5^ in 6-cm dishes) was performed. **(O)** Transfection of the pHRIG-AKT1 plasmid increased cell proliferation compared to the control group (shLacz) (#4 vs. #1). Transfection of the pHRIG-AKT1 plasmid into stable *BCL6*-knockdown BFTC905 cells for 24 h increased cell proliferation compared to shBCL6#1 and shBCL6#2 groups (#5 vs. #2; #6 vs. #3).** (P)** Stable knockdown of the *BCL6* genes (#2 & #3) markedly upregulated PTEN, FOXO3, CDKN1A and CDKN1B while downregulated pFOXO3(S253) protein levels. Transfection of the pHRIG-AKT1 plasmid into *BCL6*-knockdown BFTC905 cells for 24 h downregulated PTEN, FOXO3, CDKN1A and CDKN1B while upregulated pFOXO3(S253) protein levels compared to *BCL6*-knockdown groups (#5 vs. #2; #6 vs. #3). **(Q)** Transfection of the pHRIG-AKT1 plasmid into *BCL6*-knockdown BFTC905 cells for 24 h downregulated endogenous *CDKN1A* and *CDKN1B* (#4 vs. #1) whilst upregulated BCL6-suppressed *CDKN1A* and *CDKN1B* mRNA levels (#6 vs. #3). **(R)** Immunohistochemistry demonstrated that the protein expression levels of pFOXO3(S253) and pFOXO3(S294) were notably higher in high-stage compared to low-stage UBUCs (*n* = 295). **(S)** Both high pFOXO3(S253) and pFOXO3(S294) protein levels predicted worse disease-specific survival. *In vitro* experiments were performed in triplicate and results are expressed as the mean as means ± SEM. For immunoblot and immunohistochemical assays, representative images are shown. GAPDH served as a loading control for the immunoblot analysis. Statistical significance: *P < 0.05, **P < 0.01 and ***P < 0.001.

Due to the FOXO protein is also regulated by PI3K-AKT and MAPK (also known as ERK) signaling pathways at post-translational levels 23, we next examined the expression levels of interrelated proteins. Immunoblot analysis showed that *BCL6* overexpression in T24 cells downregulated FOXO3, however, upregulated pAKT1(S473), pMAPK1/3(T202/Y204), pFOXO3(S253) and pFOXO3(S294) protein levels. Knockdown of the *BCL6* gene in BFTC905 cells upregulated pMAPK1/3(T202/Y204), FOXO3 and pFOXO3(S294) while downregulated pan-AKT, pAKT1(S473) and pFOXO3(S253) protein levels (Figure 5A), suggesting that BCL6 may regulate FOXO3 through both AKT and MAPK1/3 pathways in T24 cells, while only via AKT signaling in BFTC905 cells. To find out through which pathways BCL6-suppressed cytostasis, inhibitors of epidermal growth factor receptor (EGFR), mitogen-activated protein kinase kinase 1/2 (MAP2K1/2) and PI3K-AKT were applied. The concentrations of these inhibitors were optimized initially (Supplementary Figure S8). Treatment with an inhibitor of EGFR or MAP2K1/2 in *BCL6*-overespressed T24 cells suppressed BCL6-induced cell proliferation (*P* < 0.001, *P* < 0.001; Figure 5B, 5C, Figure 5F, 5G) and downstream HRAS, pMAPK1/3(T202/Y204) and pFOXO3(S294) while upregulated BCL6-repressed CDKN1A, CDKN1B protein (Figure 5D, Figure 5H) and BCL6-supressed* CDKN1A* and *CDKN1B* mRNA (*P* < 0.01 to *P* < 0.001; Figure 5E, Figure 5I) levels. Likewise, treatment with a PI3K-AKT inhibitor in *BCL6*-overespressed T24 cells (Figure 5J), suppressed BCL6-induced cell proliferation (*P* < 0.001; Figure 5K), downregulated BCL6-upregulated pAKT1(S473), pFOXO3(S253) while upregulated BCL6-suppressed CDKN1A, CDKN1B protein (Figure 5L), *CDKN1A* and *CDKN1B* mRNA (*P* < 0.001; Figure 5M) abundance. Accordingly, BCL6 might regulate cell proliferation via EGFR-MAP2K-MAPK as well as PI3K-AKT signaling pathways in T24 cells. Alternatively, overexpression of the constitutively active *AKT1* gene in BFTC905 cells (Figure 5N) increased *BCL6* knockdown-suppressed cell proliferation (*P* < 0.001, *P* < 0.001; Figure 5O), upregulated pFOXO3(S253) while downregulated CDKN1A, CDKN1B protein (Figure 5P) and their corresponding mRNA (*P* < 0.01 to *P* < 0.001; Figure 5Q) levels, suggesting that BCL6 promotes cell proliferation may through modulation of the PI3K-AKT signaling pathway in BFTC905 cells. Knockdown of the *BCL6* gene notably upregulated PTEN with or without AKT1 overexpression (Figure 5P). However, quantitative RT-PCR found that BCL6 overexpression was not able to consistently downregulate *PTEN* mRNA levels in different cell lines (Supplementary Figure S9), excluded that *PTEN* as a direct transcriptional target of BCL6 in UBUC-derived cells.

Additional immunohistochemistry showed that high pFOXO3(S253) and pFOXO3(S294) protein levels correlated with high BCL6 protein level (Supplementary Table S2) and high-stage UBUC patients (Figure 5R). High pFOXO3(S253) (*P* < 0.01) and pFOXO3(294) (*P* < 0.01) protein levels conferred to poor disease-specific survival (Figure 5S). Therefore, *in vitro* experiments and clinical correlation in 295 patients suggested that BCL6 enhances FOXO3 phosphorylation and phosphorylated-FOXO3 predicted worse survival rates.

## Discussion

In this study, we initially performed array-based comparative genomic hybridization (aCGH) assays on UBUC specimens to search for candidate oncogenes pertinent to advanced UBUC. Chromosome 3q was found to be one of the most significantly gained region in UBUC patients with adverse outcomes. Across chromosome 3q, amplification of 3q27.3 was notably relevant to the muscle-invasive UBUCs. In this region, we focused on *BCL6* to evaluate its clinical and biological implications, since upregulation of *BCL6* mRNA were significantly correlated to high pT status in UBUCs patients. Amplification of the *BCL6* gene accounted for 38.1% in muscle-invasive compared to 0% in non-muscle-invasive UBUCs, which is slightly lower than those (43.8%, muscle-invasive UBUCs, *n* = 86) deposited in the TCGA database (Supplementary Figure S10). Thus our finding regarding to BCL6 amplification is about average.

We found that BCL6-suppressed cytostasis in UBUC is mediated by downregulation of *FOXO3* mRNA and its protein levels. The direct indications are that (1) BCL6 is a direct transrespressor of the *FOXO3* gene; (2) exogenous BCL6 expression downregulated *FOXO3* mRNA and its corresponding protein levels while enhanced cell proliferation; (3) knockdown of the *BCL6* gene upregulated *FOXO3* mRNA and its corresponding protein levels whereas inhibited cell proliferation; (4) simultaneously exogenous BCL6 and FOXO3 expression showed similar cell proliferation rates to the control. Among 4 mammalian FOXO members (FOXO1, -3, -4, -6), FOXO3 has a predominant role in controlling cancer development 24. Dysregulation of FOXO3 in different types of cancer including UBUC has been reported 25. Upregulation of FOXO3 inhibited bladder cancer proliferation *in vitro* and *in vivo* 26. In ER-positive breast cancer MCF-7 cells, overexpression of FOXO3 upregulates the expression of several CKIs: CDKN1A, CDKN1B and CDKN1C, which results in the regression of the growth and survival of MCF-7 cells 27. As transcription factors, FOXOs bind to the same consensus DNA sequence, the Forkhead Response Element in target genes to control transcription including a series of genes regulating cell cycle, apoptosis, autophagy, oxidative stress resistance and DNA repair, metabolism, immune-regulation and muscle atrophy (reviewed in 28). Therefore, in addition to earlier studies reported that BCL6 directly transrepressed several DNA damage and proliferation checkpoints containing *ATR serine/threonine kinase* (*ATR*), *checkpoint kinases* (*CHEKs*), *TP53*, *CDKN1A*, *CDKN2A*, *CDKN2B*, *PTEN* and others (reviewed in 29), in this study, we further found that BCL6 repressed *FOXO3* transcription and subsequent translation to promote cell proliferation in UBUC-derived cells. Although knockdown of the *BCL6* gene with 2 distinct shRNAi clones in BCTC905 cells notably upregulated PTEN protein levels, exogenous *BCL6*-expression was not able to consistently downregulated *PTEN* mRNA in 4 different UBUC-derived cell lines, suggesting that BCL6 transrepresses *FOXO3*, rather than *PTEN*, played a crucial role in *BCL6* knockdown-induced cytostasis in UBUC-derived cells. Indeed, BCL6 bound to both distinct and common sets of functionally related gene in normal GC cells versus diffuse large B-cell lymphomas-derived cells 30. Hence, BCL6 transrepressed *FOXO3* is cellular/genetic context-dependent. Intriguingly, *FOXO3* promoter activity containing Site 1 and Site 2 (-1100 to +100 bp) were much lower than those containing only Site 2 (-560 to +100 bp) with or without overexpression of the *BCL6* gene, probably due to more transcriptional repressors-binding sites, including BCL6, locate in this region (-1100 to -560 bp), responded to endogenous repressor and BCL6 protein. However, promoter activities were significantly downregulated after BCL6 overexpression in both constructs, indicating BCL6-responsive elements indeed exist in both regions.

Conversely, FOXO3 transactivates *BCL6* in the breakpoint cluster region (BCR)-Abelson proto-oncogene (ABL+) cell line, BV173 31. FOXO3 is also a specific upstream transactivator of *BCL6* in chronic myelogenous leukemia 32. We alternatively identified that BCL6 directly and negatively regulated *FOXO3* transcription and translation, suggesting that BCL6 and FOXO3 might form a negative feedback loop through AKT in the maintenance of cellular homeostasis through regulation of the cell cycle progression. *In vivo*, *BCL6*-knockdown J82 cells in a mouse xenograft model supports its oncogenic roles. *In vitro*, BCL6 negatively regulated the mRNA levels of* FOXO3*, a series of *CKIs*, their corresponding protein levels and correlated to cell proliferation. Downregulation of CKIs by BCL6 protein is frequently reported in lymphocytes, normal germinal center B and diffuse large B cell lymphomas-derived cells 33. It has been demonstrated that BCL6 interacts with the transcription factor *zinc finger and BTB domain containing 17* (ZBTB17) to suppress the transcription of the *CDKN1A* gene and cell cycle progression in germinal center B cells 34. Another possibility is that BCL6 suppressed *TP53* and next inactivated *CDKN1A* transcription and translation, since *CDKN1A* is a transcriptional target of TP53 35.

We additionally uncovered that BCL6-suppressed FOXO3 activity at the post-translational level was mediated by activation of MAPK and/or PI3K-AKT signaling pathways in distinct UBUC-derived cell lines. Indeed, the precise regulation of FOXO3 transactivation of target genes is accomplished through post-translational modifications (PTMs) and specific protein-protein interactions. Various classes of PTMs including phosphorylation operate crucial parts in directing its subcellular localization and transcription. Quite a few kinases have been reported to phosphorylate FOXO3 at specific residues and cause FOXO3 inhibition/degradation in cytoplasm or transactivation/activation in nucleus (reviewed in 36). It is well known that AKT1 phosphorylate T32, S253 and S315 whilst MAPK1/3 phosphorylates S284, S294, S325, S425 and T487 residues on the FOXO3 protein (reviewed in 37). In glioblastoma cells, knockdown of the *BCL6* gene inhibited malignant phenotype and enhanced sensitivity to temozolomide through inhibition of the AKT pathway 38. We applied EGFR, MAP2K1/2 and PI3K/AKT inhibitors in *BCL6*-overexpressed, transfection of pFOXO3(TM)-HaloTag in stable *BCL6*-knockdown cells, constitutively active AKT1 expression in *BCL6*-knockdown UBUC-derived cells in conjunction with cell proliferation and immunblot assays to provide solid evidence that BCL6 promotes cell proliferation may also through modulations of the EGFR-MAP2K1/2-FOXO3 and/or PI3K-AKT-FOXO3 signaling pathways at the post-translational level in two cell lines with genetic heterogeneity.

Importantly, immunohistochemistry assay in UBUC patients reconfirmed high correlation between tumor stage and pFOXO3(S294) (MAP2K1/2 substrate) as well as tumor stage and pFOXO3(S253) (AKT1 substrate) protein level. Either pFOXO3(S294) or pFOXO3(S253) predicts inferior outcome in UBUC patients. Thus, in addition to BCL6 transrepresses *FOXO3*, BCL6 inhibits cytostasis was also mediated by phosphorylated-FOXO3. However, the effects of *FOXO3(TM)* in cytostasis, upregulation of CDKN1A, CDKN1B protein and their corresponding mRNA levels were similar to those of the *FOXO3* gene in BCL6-overexpessed T24 cells (*P* > 0.05), indicating the *FOXO3* transcription might plays predominant roles compared to PTMs in UBUC-derived cells.

Although not widely surveyed, increased BCL6 protein levels were observed in solid tumors, implying that the tumorigenicity of BCL6 is not restricted to lymphomas. Prominently, we found that *BCL6* gene amplification in substantial muscle-invasive UBUCs. Amplifications of the *BCL6* gene were similarly found in follicular lymphoma 39, esophageal cancer 40, pancreatic tumor 41 and glioma 42. Accordingly, our studies confirmed that amplification of the *BCL6* locus is a profound cause of *BCL6* transcription and translation in muscle-invasive UBUC patients.

Altogether, we identified that high BCL6, pFOXO3(S253) and pFOXO3(S294) protein levels are poor prognostic factors for disease-specific and metastasis-free survivals in UBUC patients. Amplicification of the *BCL6* gene was found in ~50% examined cases and is a crucial cause for overexpression of *BCL6* mRNA and protein. Xenograft model suggested that BCL6 induces tumor growth and suppresses apoptosis. *In vitro* data signified that BCL6 negatively regulated *FOXO3* at transcription, translation and post-translational levels through direct binding to *FOXO3* promoter, PI3K-AKT and/or EGFR-MAP2K1/2 signaling pathway(s). Clinical data further demonstrated high correlations between BCL6 and phosphorylated/inactive FOXO3. Clinical, *in vivo* and *in vitro* evidences support that *BCL6* is an oncogene in UBUCs.

## Figures and Tables

**Table 1:** Univariate log-rank and multivariate analyses for Disease-specific and Metastasis-free Survivals in urinary bladder urothelial carcinoma.

Parameter	Category	*n*	Disease-specific Survival					Metastasis-free Survival				
			Univariate		Multivariate			Univariate		Multivariate		
			*n*	*P* value	^1^RR	95% ^2^CI	*P* value	*n*	*P* value	RR	95% CI	*P* value
Gender				0.4446			-		0.2720			-
	Male	216	41		-	-		60		-	-	
	Female	79	11		-	-		16		-	-	
Age (years)				0.1136			-		0.6875			-
	< 65	121	17		-	-		31		-	-	
	≥ 65	174	35		-	-		45		-	-	
Primary tumor (T)				< 0.0001*			0.002*		< 0.0001*			0.020*
	Ta	84	1		1	-		4		1	-	
	T1	88	9		3.236	1.449-7.246		23		4.655	1.375-15.758	
	T2-T4	123	42		17.543	1.984-166.667		49		5.963	1.708-20.816	
Nodal metastasis				0.0002*			0.532		< 0.0001*			0.045*
	N0	266	41		1	-		61		1	-	
	N1-N2	29	11		1.252	0.618-2.534		15		1.887	1.015-3.507	
Histological grade				0.0013*			0.973		0.0007*			0.822
	Low	56	2		1	-		5		1	-	
	High	239	50		0.974	0.204-4.646		71		0.885	0.303-2.579	
Vascular invasion				0.0024*			0.179		0.0001*			0.818
	Absent	246	37		1	-		54		1	-	
	Present	49	15		0.626	0.316-1.239		22		1.074	0.586-1.968	
Perineural invasion				0.0001*			0.053		0.0007*			0.193
	Absent	275	44		1	-		66		1	-	
	Present	20	8		2.246	0.988-5.106		10		1.620	0.783-3.352	
Mitotic rate (per 10 high power fields)				< 0.0001*			0.017*		< 0.0001*			0.030*
	< 10	139	12		1	-		23		1	-	
	≥ 10	156	40		2.247	1.152-4.381		53		1.767	1.057-2.954	
BCL6 protein level				< 0.0001*			0.010*		< 0.0001*			0.032*
	Low	147	8		1	-		20		1	-	
	High	148	44		2.802	1.276-6.151		56		1.819	1.053-3.144	

## References

[1] Lichtenstein P, Holm NV, Verkasalo PK (2000). Environmental and heritable factors in the causation of cancer-analyses of cohorts of twins from Sweden, Denmark, and Finland. N Engl J Med.

[2] Hall RR, Parmar MK, Richards AB, Smith PH (1994). Proposal for changes in cystoscopic follow up of patients with bladder cancer and adjuvant intravesical chemotherapy. BMJ.

[3] Wiitjes JA, Comperat E, Cowan NC (2015). Guidelines on Muscle-invasive and Metastatic Bladder Cancer. In.

[4] Shipley WU, Kaufman DS, Tester WJ (2003). Overview of bladder cancer trials in the Radiation Therapy Oncology Group. Cancer.

[5] Urbanowicz RJ, Andrew AS, Karagas MR, Moore JH (2013). Role of genetic heterogeneity and epistasis in bladder cancer susceptibility and outcome: a learning classifier system approach. J Am Med Inform Assoc.

[6] Cumberbatch K, He T, Thorogood Z, Gartrell BA (2017). Emerging drugs for urothelial (bladder) cancer. Expert Opin Emerg Drugs.

[7] Baron BW, Nucifora G, McCabe N (1993). Identification of the gene associated with the recurring chromosomal translocations t(3;14)(q27;q32) and t(3;22)(q27;q11) in B-cell lymphomas. Proc Natl Acad Sci U S A.

[8] Albagli-Curiel O (2003). Ambivalent role of BCL6 in cell survival and transformation. Oncogene.

[9] Cardenas MG, Oswald E, Yu W (2017). The Expanding Role of the BCL6 Oncoprotein as a Cancer Therapeutic Target. Clin Cancer Res.

[10] Ahmad KF, Melnick A, Lax S (2003). Mechanism of SMRT corepressor recruitment by the BCL6 BTB domain. Mol Cell.

[11] Ghetu AF, Corcoran CM, Cerchietti L (2008). Structure of a BCOR corepressor peptide in complex with the BCL6 BTB domain dimer. Mol Cell.

[12] Huang C, Gonzalez DG, Cote CM (2014). The BCL6 RD2 domain governs commitment of activated B cells to form germinal centers. Cell Rep.

[13] Kanazawa N, Moriyama M, Onizuka T (1997). Expression of bcl-6 protein in normal skin and epidermal neoplasms. Pathol Int.

[14] Lin Z, Kim H, Park H (2003). The expression of bcl-2 and bcl-6 protein in normal and malignant transitional epithelium. Urol Res.

[15] Wu Q, Liu X, Yan H (2014). B-cell lymphoma 6 protein stimulates oncogenicity of human breast cancer cells. BMC Cancer.

[16] Liang PI, Li CF, Chen LT (2014). BCL6 overexpression is associated with decreased p19 ARF expression and confers an independent prognosticator in gallbladder carcinoma. Tumour Biol.

[17] Wang YQ, Xu MD, Weng WW (2015). BCL6 is a negative prognostic factor and exhibits pro-oncogenic activity in ovarian cancer. Am J Cancer Res.

[18] Li CF, Wu WJ, Wu WR (2015). The cAMP responsive element binding protein 1 transactivates epithelial membrane protein 2, a potential tumor suppressor in the urinary bladder urothelial carcinoma. Oncotarget.

[19] Li CF, Wu WR, Chan TC (2017). Transmembrane and Coiled-Coil Domain 1 Impairs the AKT Signaling Pathway in Urinary Bladder Urothelial Carcinoma: A Characterization of a Tumor Suppressor. Clin Cancer Res.

[20] Wang YH, Wu WJ, Wang WJ (2015). CEBPD amplification and overexpression in urothelial carcinoma: a driver of tumor metastasis indicating adverse prognosis. Oncotarget.

[21] Brunet A, Bonni A, Zigmond MJ (1999). Akt promotes cell survival by phosphorylating and inhibiting a Forkhead transcription factor. Cell.

[22] Dreos R, Ambrosini G, Groux R (2017). The eukaryotic promoter database in its 30th year: focus on non-vertebrate organisms. Nucleic Acids Res.

[23] Farhan M, Wang H, Gaur U (2017). FOXO Signaling Pathways as Therapeutic Targets in Cancer. Int J Biol Sci.

[24] Lam EW, Brosens JJ, Gomes AR, Koo CY (2013). Forkhead box proteins: tuning forks for transcriptional harmony. Nat Rev Cancer.

[25] Liu Y, Ao X, Ding W (2018). Critical role of FOXO3a in carcinogenesis. Mol Cancer.

[26] Yu C, Zhang Z, Liao W (2012). The tumor-suppressor gene Nkx2.8 suppresses bladder cancer proliferation through upregulation of FOXO3a and inhibition of the MEK/ERK signaling pathway. Carcinogenesis.

[27] Zou Y, Tsai WB, Cheng CJ (2008). Forkhead box transcription factor FOXO3a suppresses estrogen-dependent breast cancer cell proliferation and tumorigenesis. Breast Cancer Res.

[28] van der Vos KE, Coffer PJ (2011). The extending network of FOXO transcriptional target genes. Antioxid Redox Signal.

[29] Hatzi K, Melnick A (2014). Breaking bad in the germinal center: how deregulation of BCL6 contributes to lymphomagenesis. Trends Mol Med.

[30] Adjuvant chemotherapy in invasive bladder cancer (2005). a systematic review and meta-analysis of individual patient data Advanced Bladder Cancer (ABC) Meta-analysis Collaboration. Eur Urol.

[31] Fernandez de Mattos S, Essafi A, Soeiro I (2004). FoxO3a and BCR-ABL regulate cyclin D2 transcription through a STAT5/BCL6-dependent mechanism. Mol Cell Biol.

[32] Hurtz C, Hatzi K, Cerchietti L (2011). BCL6-mediated repression of p53 is critical for leukemia stem cell survival in chronic myeloid leukemia. J Exp Med.

[33] Shaffer AL, Yu X, He Y (2000). BCL-6 represses genes that function in lymphocyte differentiation, inflammation, and cell cycle control. Immunity.

[34] Phan RT, Saito M, Basso K (2005). BCL6 interacts with the transcription factor Miz-1 to suppress the cyclin-dependent kinase inhibitor p21 and cell cycle arrest in germinal center B cells. Nat Immunol.

[35] Fischer M (2017). Census and evaluation of p53 target genes.

[36] Wang X, Hu S, Liu L (2017). Phosphorylation and acetylation modifications of FOXO3a: Independently or synergistically?. Oncol Lett.

[37] Zhao Y, Wang Y, Zhu WG (2011). Applications of post-translational modifications of FoxO family proteins in biological functions. J Mol Cell Biol.

[38] Song W, Wang Z, Kan P (2018). Knockdown of BCL6 Inhibited Malignant Phenotype and Enhanced Sensitivity of Glioblastoma Cells to TMZ through AKT Pathway. Biomed Res Int.

[39] Karube K, Ying G, Tagawa H (2008). BCL6 gene amplification/3q27 gain is associated with unique clinicopathological characteristics among follicular lymphoma without BCL2 gene translocation. Mod Pathol.

[40] Chen J, Guo L, Peiffer DA (2008). Genomic profiling of 766 cancer-related genes in archived esophageal normal and carcinoma tissues. Int J Cancer.

[41] Holzmann K, Kohlhammer H, Schwaenen C (2004). Genomic DNA-chip hybridization reveals a higher incidence of genomic amplifications in pancreatic cancer than conventional comparative genomic hybridization and leads to the identification of novel candidate genes. Cancer Res.

[42] Xu L, Chen Y, Dutra-Clarke M (2017). BCL6 promotes glioma and serves as a therapeutic target. Proc Natl Acad Sci U S A.

