# Supplementary material for: Amplification-driven BCL6-suppressed cytostasis is mediated by transrepression of FOXO3 and post-translational modifications of FOXO3 in urinary bladder urothelial carcinoma
Source: Theranostics. 2020 Jan 1;10(2):707–24. doi: 10.7150/thno.39018 (PMC6929993; doi:10.7150/thno.39018)
Supplement: Supplementary file 1 — Supplementary figures and tables. [file thnov10p0707s1.pdf]

Supplementary Document

Supplementary Methods

Patients and specimens

Patients with confirmed or suspected lymph node metastasis received regional lymph node dissection. Cisplatin-based post-operative adjuvant chemotherapy was performed in those with pT3, pT4 status or nodal involvement. The histological diagnosis of urinary bladder urothelial carcinoma (UBUC) was confirmed in all cases based on the latest World Health Organization classification. Histological grading was based on Edmonson and Steiner's criteria, while tumor staging was determined according to the 7th edition of the American Joint Committee on Cancer system. Medical charts were reviewed for each patient to ascertain the accuracy of other pertinent clinicopathological data. Follow-up information was available in all cases with a median period of 42 months (ranging 3-176 months).

Quantitative RT-PCR and quantitative PCR

Quantitative RT-PCR assay was applied to quantify the expression levels of several transcript using predesigned TaqMan® assay reagents [*BCL6*: Hs00153368_m1, 76 bp, NM_001130845.1; cyclin-dependent kinase inhibitor 1A (*CDKN1A*, i.e., *p21*): Hs00355782_m1, 66 bp, NM_000389.4; *CDKN1B* (i.e., *p27*): Hs01597588_m1, 151 bp; NM_004064.3, *CDKN1C* (i.e., *p57*): Hs00175938_m1, 91 bp, NM_000076.2; *CDKN2D* (i.e., *p19*): Hs. 00176481_m1, 52 bp; Tumor protein p53 (*TP53*): Hs01034249_m1, 111 bp; Forkhead box O3 (*FOXO3*): Hs00818121_m1, 214 bp; and glyceraldehyde-3-phosphate dehydrogenase (*GAPDH*): Hs03929097_g1, 58 bp, NM_001256799.1 (internal control)] (ThermoFisher Scientific), LightCycler® 96 Real-Time PCR System and $\Delta\Delta C_T$ calculation. Briefly, total RNAs were extracted with TRIzol® reagent (ThermoFisher Scientific) from cells and reverse-transcribed using the High Capacity cDNA Reverse Transcription Kit (ThermoFisher Scientific). The relative expression fold of each transcript was given by $2^{-\Delta\Delta CT}$, where $\Delta\Delta C_T = \Delta C_T$ ($_{\text{Ta/T1 cells, T2-T4 cells, 3q27 amplification, 3q27 non-amplification, transfected cells, knockdown cells etc.}}$) - ΔC_T ($_{\text{HUC or control}}$); ΔC_T represented the C_T of a target transcript subtracted the C_T of *GAPDH* for each specimen or cell line. Only samples with C_T values < 28 for *GAPDH* were considered to meet acceptable RNA quality standards and included in the analyses. For ChIP assay to amplify potential BCL6 responsive elements, primers and probes targeting site 1 (primers: 5'-GGCACGGATCGTAGAATAAGGA-3' & 5'-GCTACGTGGGCATTTTTGACTT-3'; probe: 5'-AAAGGATACGTCTTTT-3') and site 2 (5'-TCAGTATTTCCACACCGCGA-3', 5'-CGGTGTCCGGTTCCCTG-3'; probe: 5'-TCGCGTTCTAACAGG-3') were used.

Immunohistochemistry

Slides were deparaffinized with xylene, rehydrated with ethanol, heated by microwave for retrieval of antigen epitopes in a 10 mM citrate buffer (pH 6) for 7 min. Endogenous peroxidase was quenched by 3% H_2O_2. Slides were washed with Tris-buffered saline for 15 min and then incubated with a primary monoclonal antibody against BCL6 (1:20; sc-858, Santa Cruz, Santa Cruz, CA, USA), Ki-67 (1:200, abcam, Cambridge, UK), terminal deoxynucleotidyl transferase dUTP nick end labeling (TUNEL) (Roche, Basel, Switzerland), pFOXO3(S294) (1:50, #5538S, Cell Signaling, Danvers, MA, USA) and pFOXO3(S253) (1:25, ab47285, Abcam) for 1 h, followed by antibody detection using a ChemMate EnVision™ kit (K5001; DAKO, Agilent, Santa Clara, CA, USA).

Cell culture
Human normal urothelial cells (HUC; #4320, ScienCell, Carlsbad, CA, USA) were obtained and cultured with recommended medium in the poly-L-lysine coated flask (2 μg/cm^2). The human UBUC-derived cell lines, RT4 (ATCC, Manassas, VA, USA), BFTC905 [Bioresource Collection and Research Center (BCRC), Hsinchu, Taiwan], J82 (ATCC), renal pelvis carcinoma-derived BFTC909 (BCRC) and T24 (ATCC) cells were maintained in DMEM supplemented with 10-15% (v/v) fetal bovine serum (Biological Industries), appropriate nutrients and antibiotics in a humidified incubator with 5% CO_2 at 37°C. Media were obtained from CORNING. BFTC905, BFTC909 and J82 cells were all characterized as stage T3 (Tzeng et al.1996; *Anticancer Res.* 16:1797-804; O'Toole et al. 1978; *Br. J. Cancer*, 38:64-76). All cell lines were authenticated by short tandem repeat genotyping, periodically confirmed to be mycoplasma-free using PlasmoTest™ (ThermoFisher, Waltham, MA, USA).

Lentivirus production and stable knockdown of the *BCL6* and *FOXO3* genes
Clones were obtained from the National RNAi Core Facility, Institute of Molecular Biology, Academia Sinica, Taipei, Taiwan. A total of 4 plasmids targeting the *BLC6* gene were preliminarily screened. The *BCL6* mRNA levels could be effectively downregulated by only 2 clones. Another two shFOXO3 clones were also identified. Briefly, Phoenix-AMPHO cells (ATCC) were seeded in 6-cm tissue culture plate at a density of 3 × 10^6 in 5 mL medium with 10% FBS, 100 IU/mL penicillin and 100 μg/mL streptomycin (Corning®, Corning, NY, USA) overnight. PolyJet™ (15 μL, #SL100688, SignaGen® Laboratories, Rockville, MD, USA) was used to transfect the plasmid mixture [psPAX2 (2.25 μg, Addgene), PMD2.G (0.25 μg, Addgene) and 2.5 μg of shLacZ (control), shBCL6#1 and shBCL6#2 plasmids], and the medium was changed after a 16 h incubation. Medium was collected and filtered (0.22 μm) at 40 h and 64 h post-transfection, aliquots of 1 mL were stored at -80°C for further infection. For stable shRNAi, lentiviral particles were produced and 1 × 10^6 BFTC905 and BFTC909 cells were next transduced with media containing lentiviral particles containing polybrene (8 μg/mL) and incubated for another 24 h at 37°C. Afterwards, media containing 4 μg/mL puromycin were used to select positive cells for 3 days and subsequently maintained in media containing 2 μg/mL puromycin for further experiments. The same protocol was performed in double knockdown of the *BCL6* and *FOXO3* genes in BFTC905 cells.

Immunoblot analysis
Cell lysates were prepared with RadioImmunoPrecipitation Assay buffer (Merck Millipore, Burlington, MA, USA). Lysates containing equal amounts of protein were separated by SDS-PAGE and electroblotted onto FluoroTrans® PVDF Transfer Membrane (PALL). The filters were individually probed with specific primary antibody. Protein bands were detected by the Western Lightning Chemiluminescence Reagent Plus Kit (Perkin-Elmer Life Sciences, Cleveland, OH, USA) with horseradish peroxide labeled secondary antibody as suggested by the manufacturer and visualized on a ChemiDoc™ XRS+ System (Bio-Rad, Hercules, CA, USA). The intensity of bands was quantified by densitometry (ImageJ) and normalized to glyceraldehyde-3-phosphate dehydrogenase (GAPDH) or pan-actin in each lane.

Anti-human BCL6 transcription repressor (BCL6, 1:500, sc-858, Santa Cruz), HaloTag (1:1000, G9211, Promega), tumor protein p53 (TP53, 1:1000, #2524, Cell Signaling), pTP53(S15) (1:1000, #9286, Cell Signaling), pTP53(S20) (1:1000, #9287, Cell Signaling), cyclin-dependent kinase inhibitor 1A (CDKN1A, 1:1000, #2947, Cell Signaling), CDKN1B (1:500, #3686, Cell Signaling), CDKN1C (1:500, sc-1040, Santa Cruz), CDKN2B (1:500, sc-612, Santa Cruz), CDKN2C (1:500, sc-1208, Santa Cruz), CDKN2D (1:500, sc-1063, Santa Cruz), cyclin D1 (CCND1, 1:500, #2926, Cell Signaling), cyclin-dependent kinase 4

(CDK4, 1:500, sc-260, Santa Cruz), CCNE1 (1:500, sc-198, Santa Cruz), CDK2 (1: 500, sc-163, Santa Cruz), RB transcriptional corepressor 1 (RB1, 1:500, sc-102, Santa Cruz), E2F transcription factor 1 (E2F1, 1:500, sc-251, Santa Cruz), transcription factor Dp-1 (TFDP1, 1:500, sc-53642, Santa Cruz), AKT serine/threonine kinase (pan-AKT, 1:1000, #4691, Cell Signaling), pAKT1(S473) (#4060, Cell Signaling), phosphatase and tensin homolog (PTEN, 1:1000, GTX101025, GeneTex, Irvine, CA, USA), mitogen-activated protein kinase 1 (MAPK1/3, 1:1000, #4695, Cell Signaling), pMAPK1/3(T202/Y204) (1:1000, #4370, Cell Signaling), forkhead box O3 (FOXO3, 1:1000, ab12162, Abcam), pFOXO3(S253) (1:1000, #9466, Cell Signaling), pFOXO3(S294) (1:1000, #5538, Cell Signaling) and HRas proto-oncogene, GTPase (HRAS, 1:1000, GTX116041, GeneTex) were used as primary antibodies for immunoblotting analysis and anti-GAPDH (1:5000, ab128915, Abcam) served as a loading control.

Cell-cycle, 5-bromo-2'-deoxyuridine, cell proliferation, soft agar/anchorage-independent growth

For cell cycle analysis, 1×10^6 cells were collected, washed with ice-cold PBS, fixed with 70% ethanol and stored at -20°C after stable transfection of pCMV6-Entry, pCMV6-BCL6 plasmid, or infection with shBCL6#1, shBCL6#2 or shLacZ lentiviral particles. Before analysis, fixed cells were washed with ice-cold PBS for three times and treatments with 200 μg/mL RNase A and 20 μg/mL propidium iodide (PI). A total of 10,000 events were analyzed; cell cycle distribution was analyzed by a Beckman Coulter Epics XL Flow Cytometer and the Modfit LT™ software (BD Biosciences, Franklin Lakes, NJ, USA) (Kuo et al. 2011; *Toxicol Appl Pharmacol* 256:8-23).

To determine cell proliferation upon alternation of BCL6 expression levels, 3×10^3 cells were seeded on 96-well microplates for 5-bromo-2'-deoxyuridine (BrdU) or cell proliferation (fluorometric) (#K307-1000, Biovision, Milpitas, CA, USA) assays. After removing the medium, BrdU Cell Proliferation Assay Kit (QIA58, Merck Millipore) was used to perform cell proliferation test. BrdU label (1:2000 dilution) was incubated for 24 h. Plates were then washed, stained with anti-BrdU antibody, and peroxidase-conjugated goat anti-mouse IgG. 3,3’,5,5’-tetramethylbenzidine substrate (0.1 mL in ethanol) was next added into the immunocomplex and the reaction was terminated via adding 100 μL of sulfuric acid (2.5 N). Absorbances were afterward measured at wavelengths 450/540 nm using a Beckman Coulter PARADIGM™ Detection Platform. Percentages of proliferation rate (%) were calculated as $100 \times [(OD_{\text{indicated time after transfection}} - OD_{\text{7d after transfection}})/OD_{\text{7d after transfection}}]$. Experiments were triplicated and results are expressed as mean ± SEM. On the other hand, Biovision’s Cell Proliferation Assay Kit is based on a nuclear dye that specifically binds nucleic acid in the cell and generates green fluoresces. The generated fluorescent intensity is directly proportional to the cell number, which can be quantified by measuring fluorescence (Ex/Em = 480/538 nm).

CytoSelect™ 96-well in vitro tumor sensitivity assay (soft agar colony formation, CBA-150-5, Cell Biolabs, Inc., San Diego, CA, USA) was used to analyze whether stable expression and knockdown of the *BCL6* gene affected anchorage-independent cell growth. Briefly, 50 μL/well (in a 96-well sterile flat-bottom microplate) of the Base Agar Matrix Layer was prepared by mixing 1.25 mL of 2X DMEM/20% FBS medium, 1 mL of sterile water, 0.25 mL of melted 10X CytoSelect™ Agar Matrix Solution. Cell Suspension/Agar Matrix Layer under sterile conditions (75 μL/well) was made by mixing 1.75 mL of 2X DMEM/20% FBS medium, 1.375 mL of CytoSelect™ Matrix Diluent, 0.375 mL of melted 10X CytoSelect™ Agar Matrix Solution and 0.25 mL of Cell Suspension (5×10^3 cells), according to the manufactures’ instructions. The incubation periods were 8 days for both

BCL6-overexpressed J82 and *BCL6*-knockdown BFTC905 and BFTC909 cells. 3-(4,5-dimethylthiazol-2-yl)-2,5-diphenyltetrazolium bromide (MTT) assay was used to quantitate the anchorage-independent growth.

Wound healing, transwell migration and transwell invasion

Wound healing assay and QCM ECMatrix Cell Invasion Kit (ECM554, Millipore) were used to measure cell migration and invasion. Before the cells reached confluence in 6-mm culture plates, a cell-free gap was created using a silicon Culture-Insert (ibidi GmbH) placed on the Petri dishes. After removing the silicon insert from the surface, a clean gap was formed. Cell migration into the clean region was recorded using Axiovert 40 CFL (Zeiss International) at 0, 4, 8 and 12 h, and the percentage of wound healing was determined via dividing the migrated distance by the scratched distance. For transwell invasion assay, *BCL6*–overexpressed J82 (8×10^5) and *BCL6*–knockdown BFTC905 (1×10^6) and BFTC909 cells (8×10^5) were starved in media (250 μL) containing 0.5% FBS at 37°C overnight, seeded in ECMatrix™-coated inserts in a 24-well plate. Literally 500 μL of media containing 10% (J82 and BFTC905 cells) or 15% (BFTC909 cells) FBS were added into the lower chambers and cells were cultured for another 48 h. The inserts were removed, placed into new lower chambers and the penetrated cells were detached with Cell Detachment Solution and lysed with Lysis Buffer/Dye Solution. The lysed mixtures were transferred to a 96-well plate for fluorescence measurement at wavelengths 480/520 nm using a Beckman Coulter PARADIGM™ Detection Platform. For transwell migration assay, ECMatrix™-coated inserts were replaced by BD Falcon Cell Culture Inserts (#353097).

Gelation zymography and MMP2/MMP9 assay

Gelatin zymography was performed based on an early study (Fredriksson et al. 2006; *Am J Physiol Lung Cell Mol Physiol* 290:L326-33), with some modifications. Cells (5×10^5 in 6-cm dishes) were cultured in serum-free medium (2 mL) for 24 h at 37°C with 5% CO_2 before gelatin zymography analysis. Culture media were centrifuged at 4°C, 300 × g for 10 min and the supernatants were collected. Literally 10 μL of the supernatant and 10 μL of 2X sample buffer (2% SDS, 0.1% bromophenol blue and 40% glycerol in stacking gel buffer which contains 84 mM ammediol/HCl and 0.02% NaN_3) were mixed. Samples were separated by electrophoresis on 6% SDS-polyacrylamide gels containing 0.1% gelatin without reducing agent at 4°C. After electrophoresis, gels were washed with 2.5% Triton X-100 twice on a shaker for 30 min at room temperature to remove the SDS. Gels were next incubated at 37°C for 48 h in 20 mL of development buffer (200 mM NaCl, 5 mM $CaCl_2 \cdot 2H2O$, 50 mM Tris-base, 0.02% NaN_3), washed with H_2O twice and stained with 0.1% Coomassie blue R250 dissolved in 10% acetic acid and 50% methanol in H_2O. Afterward, gels were rinsed with H_2O for 1 h. The digested areas appeared clear on a blue background, indicating the location of gelatinases.

MMP2/MMP9 assay kit *(*#E-118SA, Biomedical Research Service Center, University at Buffalo, State University of New York, USA) was used to analyze the MMP activities. MMP2/MMP9 activity is determined by optical measurement of 595 nm with a GloMax®-Multi Detection System (E7061, Promega) after acetone precipitation of undigested blue gelatin, according to the manufacturer's instructions.

Analysis of correlation between *BCL6* and *FOXO3A* transcript expression in Oncomine cohorts

Gene expression levels for *BCL6* and *FOXO3A* in 188 UBUCs was obtained from a public domain dataset (GSE13507, https://www.ncbi.nlm.nih.gov/geo/query/acc.cgi?acc=GSE13507)

by using Oncomine^{TM} Research Premium Software. The data were extracted from https://software.oncomine.com/resource/main.html#d:156636604;dso:geneOverex;dt:dataset;ec:[2];epv:150001.151078,3508;et:over;f:173327157;g:2309;p:200009673;pg:1;pvf:2941[Urothelial Carcinoma of the Urinary Bladder],5756,12811[FOXO3A];scr:datasets;ss:all;v:18.

Validation of *BCL6* copy number gain in Oncomine TCGA cohorts

The *BCL6* copy number data was obtained from 152 UBUCs in the TCGA dataset deposited at Oncomine^{TM} cohort. Of the 152 UBUCs, 86 were designated as muscle-invasive bladder cancer (MIBC) which allows further subgroup analysis. The copy number data were extracted from https://software.oncomine.com/resource/main.html#d:156636581;dso:geneOverex;dt:dataset;ec:[2,1,3];epv:150001.151078,3508,3519,4018;et:over;f:146613447;g:604;gt:barchart;p:200009125;pg:1;pvf:2941[Urothelial Carcinoma of the Urinary Bladder],3520,5756;scr:datasets;ss:all;v:18

Supplementary Tables

Table S1 Clinical pathological features of 35 UBUC samples submitted to aCGH analysis

**Parameter**		**Number**
Age (years)		
	< 60	5
	≥ 60	30
Gender		
	Male	24
	Female	11
Primary tumor status		
	Non-muscle invasive	14
	Muscle invasive	21
Nodal metastasis		
	Negative	31
	Positive	4
Histological grade		
	Low grade	2
	High grade	33

Table S2 Correlations between BCL6 protein level and clinicopathological parameters in urinary bladder urothelial carcinoma

Parameter	Category	Urinary Bladder Urothelial Carcinoma			
		n	BCL6 protein level		*P* value
			Low	High	
Gender					0.923
	Male	216	108	108	
	Female	79	39	40	
Age (years)					0.686
	< 65	121	62	59	
	≥ 65	174	85	89	
Primary tumor (T)					**< 0.001***
	Ta	84	66	18	
	T1	88	53	35	
	T2-T4 (muscle invasive)	123	28	95	
Nodal metastasis					**< 0.001***
	Negative (N0)	266	144	122	
	Positive (N1-N2)	29	3	26	
Histological grade					**< 0.001***
	Low grade	56	42	14	
	High grade	239	105	134	
Vascular invasion					**< 0.001***
	Absent	246	137	109	
	Present	49	10	39	
Perineural invasion					**0.006***
	Absent	275	143	132	
	Present	20	4	16	
Mitotic rate (per 10 high power fields)					**0.023***
	< 10	139	79	60	
	≥ 10	156	68	88	
pFOXO3(S253) protein level					**< 0.001***
	Low	222	141	81	
	High	73	6	67	
pFOXO3(S294) protein level					**< 0.001***
	Low	267	142	125	
	High	28	5	23	

*, Statistically significant

Supplementary Figures

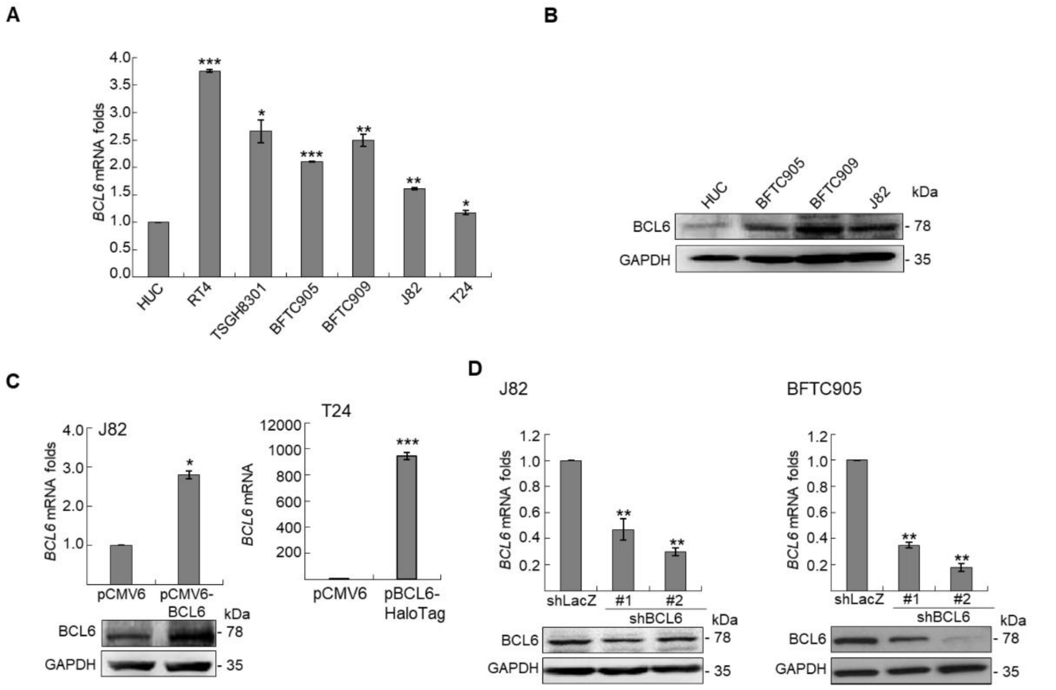

Figure S1. Stable overexpression and knockdown of the *BCL6* gene in distinct UBUC-derived cell lines notably induces and suppresses *BCL6* mRNA and protein levels. Quantitative RT-PCR and immunoblot analysis showed that **(A)** *BCL6* mRNA levels were highly expressed in distinct UBUC-derived cell lines, compared to normal human urothelial cells (HUC). **(B)** Endogenous BCL6 protein levels were highly expressed in BFTC905, BFTC909 and J82 cells, compared to that of HUC. **(C)** Stable transfection of the pCMV-BCL6 plasmid into J82 and T24 cells by selection with G418 notably upregulated *BCL6* mRNA and/or protein levels. **(D)** Stable knockdown of the *BCL6* gene with two distinct shRNAi clones (shBCL6#1 and shBCL6#2) in J82, BFTC905 and BFTC909 cells by puromycin selection markedly downregulated *BCL6* mRNA and protein levels. All experiments were performed in triplicate and results are expressed as the mean ± SEM. For immunoblot analysis, one representative image is shown and GAPDH served as a loading control. Statistical significance: *, $P < 0.05$; **, $P < 0.01$; ***, $P < 0.001$.

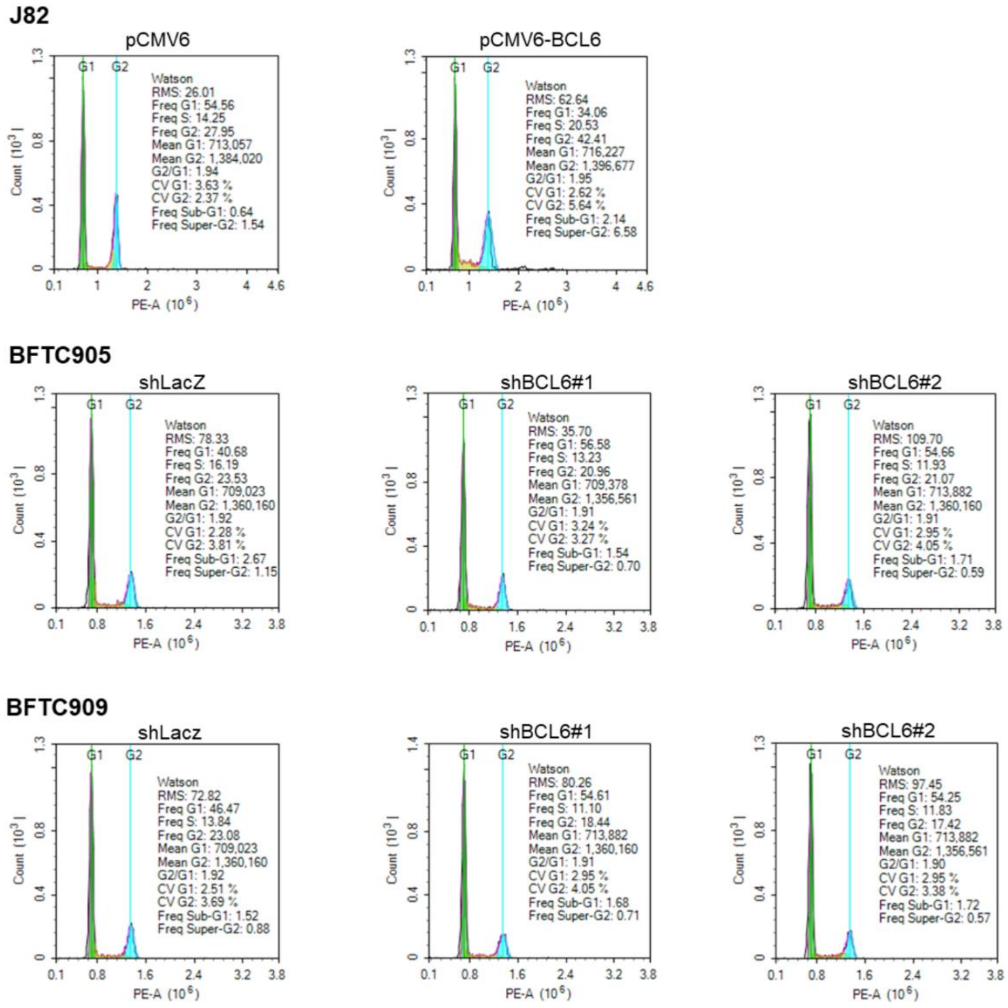

Figure S2. Overexpression of the *BCL6* gene in J82 and cells induced cell cycle progression to S and G_2/M phases. Knockdown of the *BCL6* gene in BFTC905 and BFTC909 cells showed opposite results.

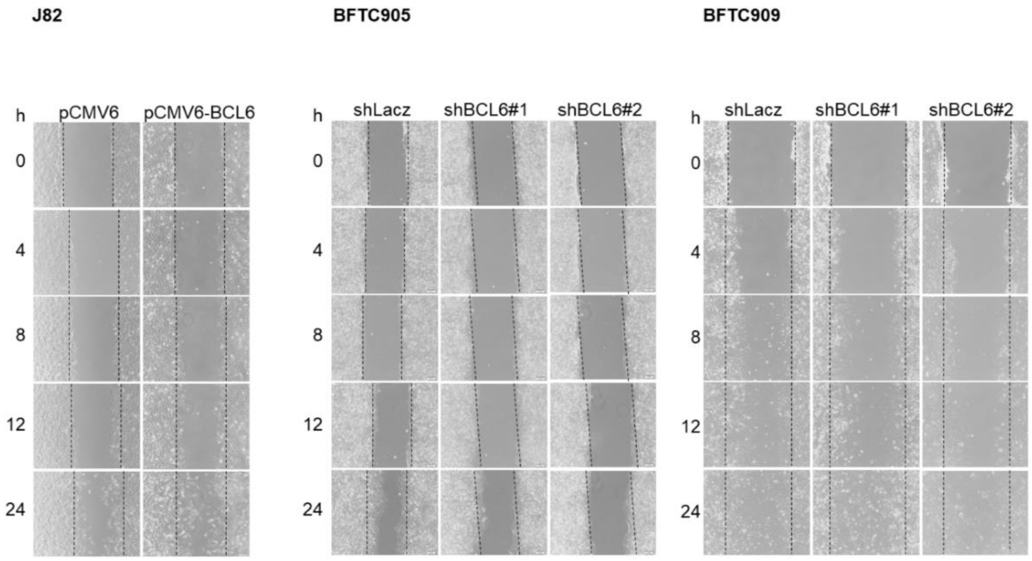

Figure S3. BCL6 induces cell migration. Wound-healing assay on stable *BCL6*-overexpressed J82 and –knockdown BFTC905 and BFTC909 cells showed escalation and suppression of cell migration.

Transwell migration assay

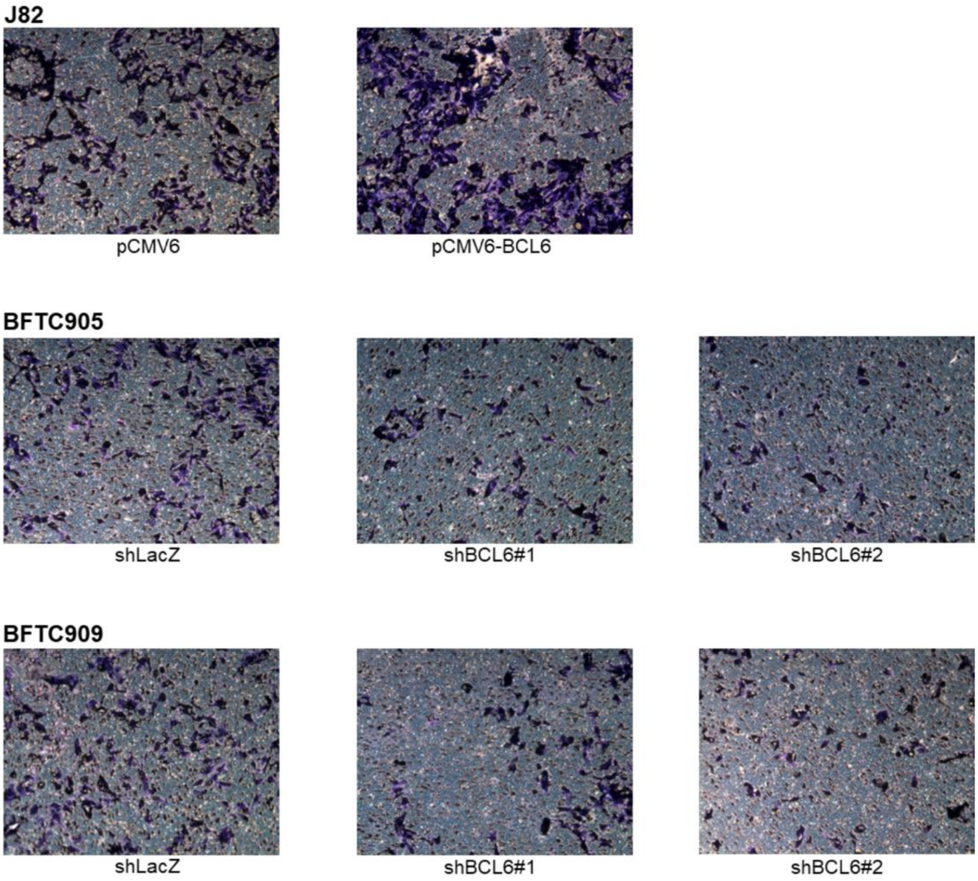

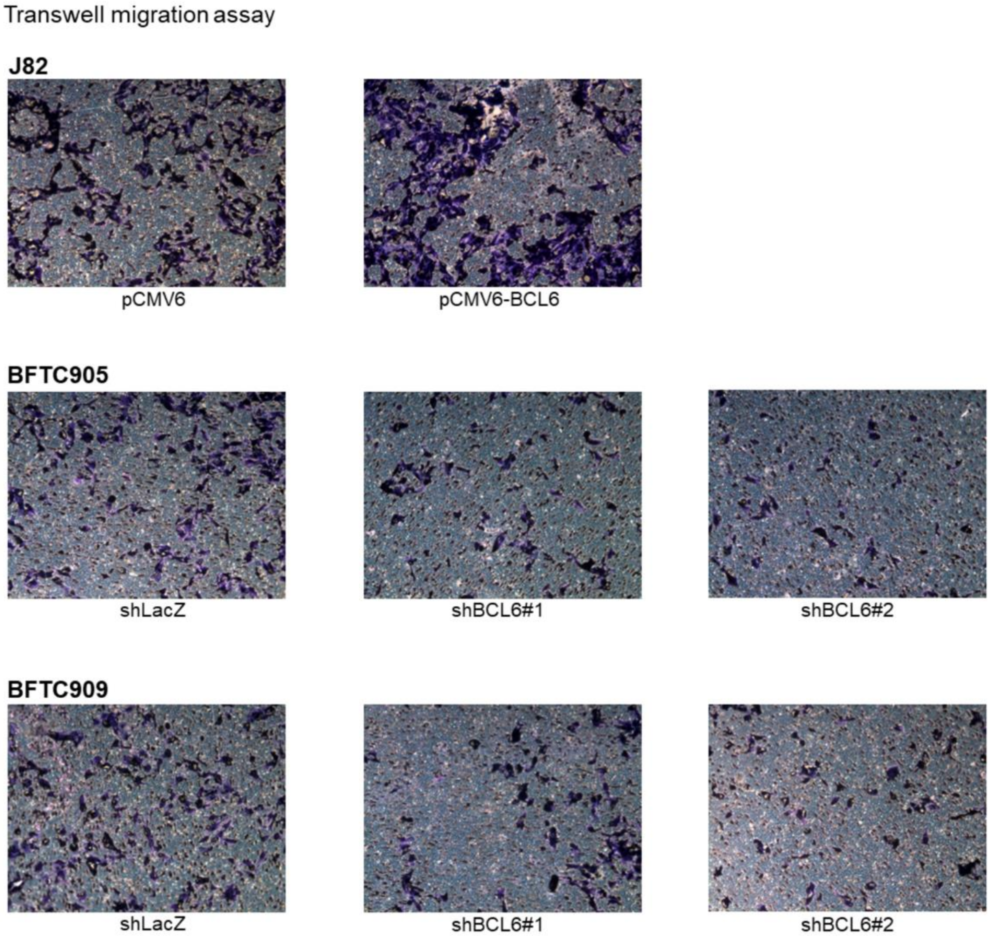

Figure S4. Transwell migration and transwell invasion assays showed that overexpression of the *BCL6* gene in J82 cells induced, while knockdown of the *BCL6* gene in BFTC905 and BFTC909 cells inhibited migration and invasion.

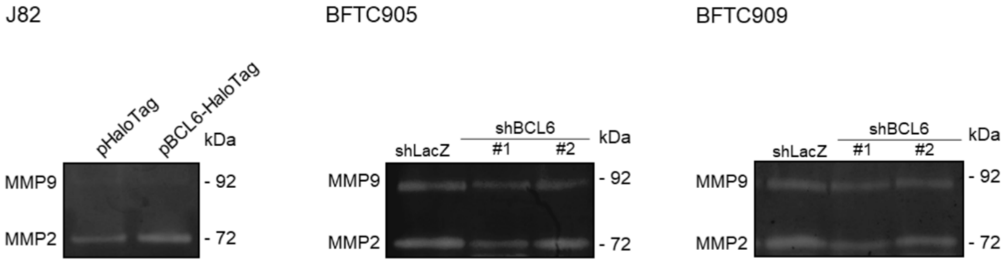

Figure S5. Gelatin zymography on stable *BCL6*-overexpressed J82 and –knockdown BFTC905 and BFTC909 cells showed MMP2 and/or MMP9 activities were increased and decreased, respectively. Experiments were performed in triplicate and representative images are shown.

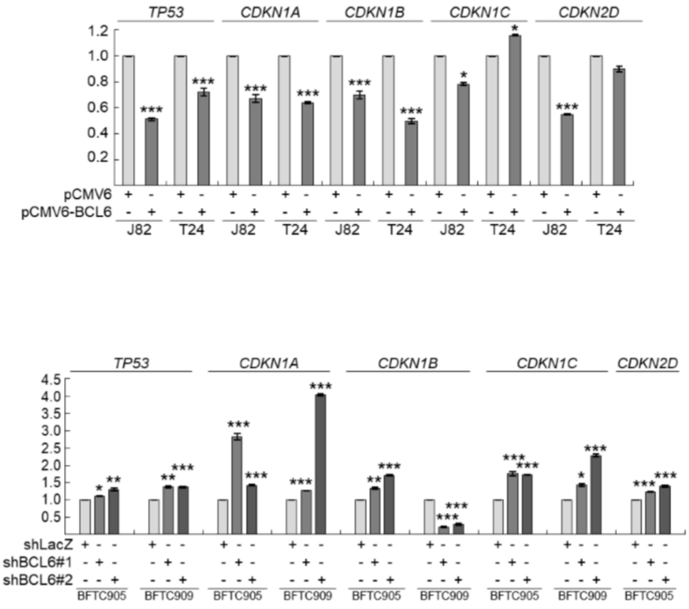

Figure S6. Stable transfection of the pCMV-BCL6 plasmid into J82 and T24 cells downregulated *TP53, CDKN1A* and *CDKN1B,* while knockdown of the *BCL6* gene with shBCL6#1 and shBCL6#2 in BFTC905 and/or BFTC909 cells upregulated *TP53, CDKN1A, CDKN1C* and *CDKN2D* mRNA levels. All experiments were performed in triplicate and results are expressed as the mean ± SEM. Statistical significance: *$P < 0.05$; **$P < 0.01$; ***$P < 0.001$.

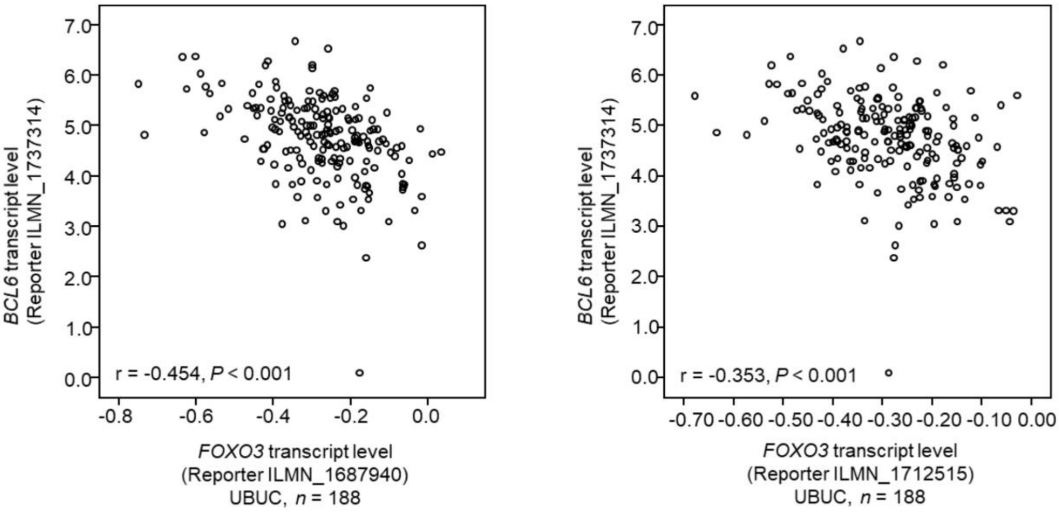

Figure S7. The correlation coefficients between *BCL6* and *FOXO3* mRNA levels with two *FOXO3* probes were -0.454 and -0.353, respectively, in GSE13507 (Gene Expression Omnibus database, NCBI).

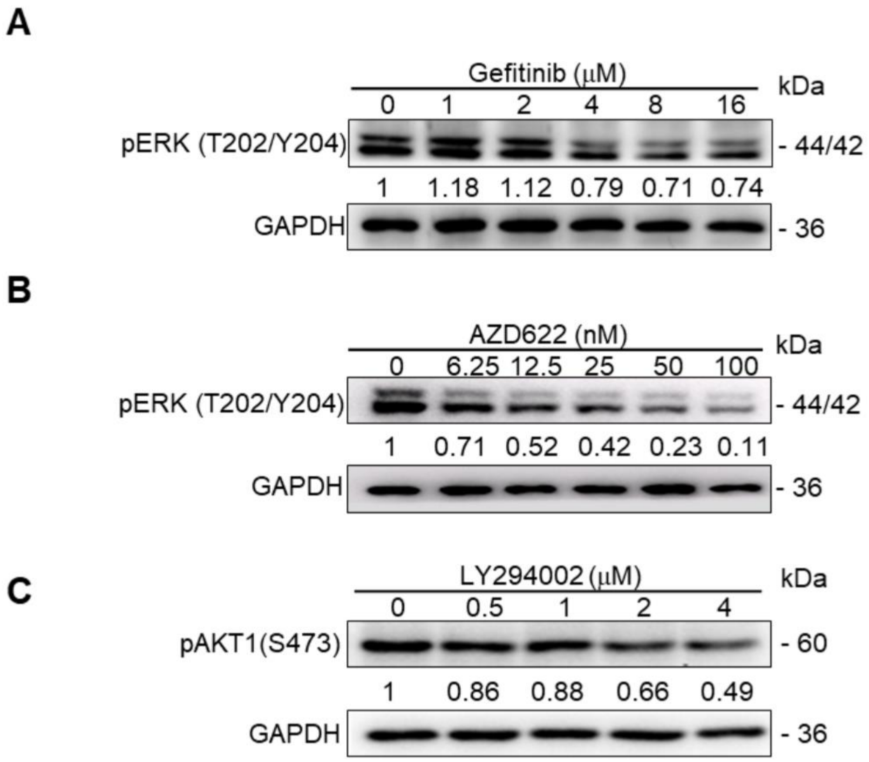

Figure S8. Dose-response experiments identified that optimal concentrations of several inhibiters after treatment for 24 h in T24 cells. **(A)** Gefitinib: 8 μM, **(B)** AZD6244: 6.25 nM and (C) LY294002: 4μM.

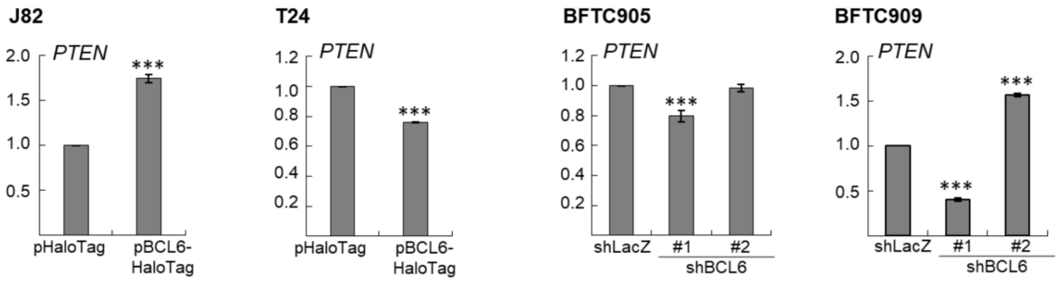

Figure S9. Stable transfection of the pCMV-BCL6 plasmid into J82 and T24 cells or knockdown of the *BCL6* gene in BFTC905 and BFTC909 cells were not able to consistently downregulate or upregulate *PTEN* mRNA levels.

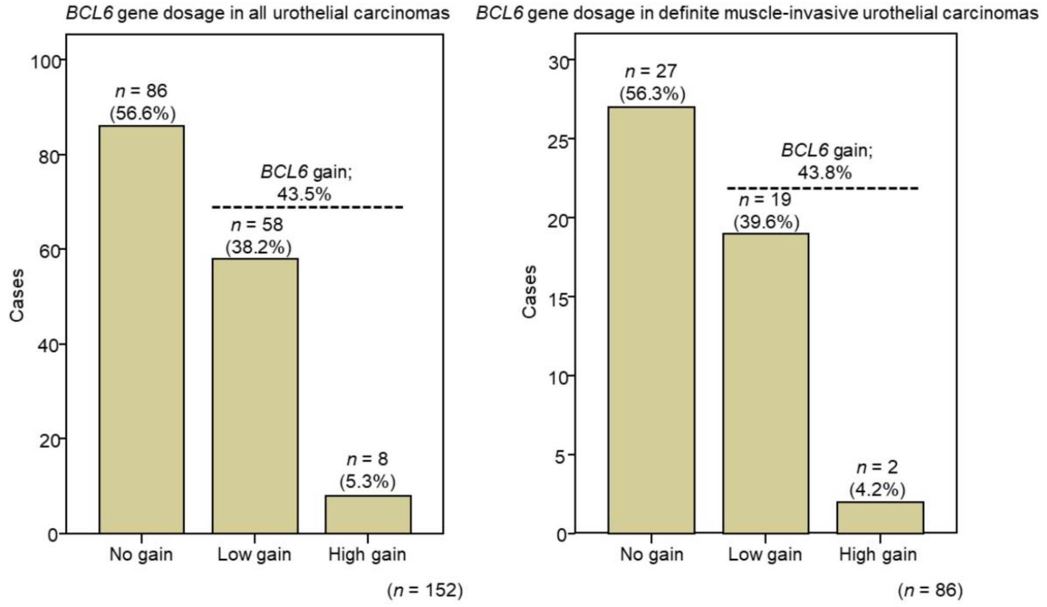

Figure S10. Amplification of the *BCL6* gene accounted for 43.5% and 43.8% in all urothelial and muscle-invasive urothelial carcinoma, respectively, from data deposited in the TCGA database.
